# HRV-Based Multimodal Physiological Signal Monitoring Using Wearable Biosensors in Human–Computer Interaction: Cognitive Load in Real-Time Strategy Games

**DOI:** 10.3390/s26072181

**Published:** 2026-04-01

**Authors:** Yunlong Shi, Muyesaier Kuerban, Yiyang Jin, Chaoyue Wang, Lu Chen

**Affiliations:** X-Laboratory of Intelligent Interaction (II X-LAB), School of Mechanical Engineering and Automation, Beihang University, 37 Xueyuan Road, Haidian District, Beijing 100191, China; shiyunlong@buaa.edu.cn (Y.S.); muyesaierkuerban@buaa.edu.cn (M.K.); jinyiyang@buaa.edu.cn (Y.J.); wanglichaoyue@buaa.edu.cn (C.W.)

**Keywords:** cognitive load, heart rate variability, wearable biosensors, NASA-TLX, real-time strategy games, human–computer interaction, multitasking, visual complexity, decision pressure

## Abstract

Real-time strategy (RTS) games provide a cognitively demanding and ecologically valid context for investigating workload dynamics in human–computer interaction (HCI). This multimodal study (HRV, NASA-TLX, behavior, interviews) examined multitasking, visual complexity, and decision pressure in 36 novice RTS players. High multitasking significantly increased subjective workload (total raw-TLX: from 22.50 ± 14.65 to 36.47 ± 20.19, *p* < 0.001) and prolonged completion time (from 317.17 ± 37.26 s to 354.92 ± 50.70 s, *p* < 0.001). Decision pressure elevated subjective workload (total raw-TLX: from 20 to 28, *p* = 0.008) without affecting performance. Although HRV did not consistently differentiate experimental conditions at the group level, it showed stable individual-level associations with perceived workload—both in expected directions (e.g., LF power positively correlated with total raw-TLX across four experiments, r = 0.28–0.53, all *p* < 0.05) and in inverse relationships that deviate from conventional stress models (e.g., stress index negatively correlated with total raw-TLX, r = −0.34 to −0.40, all *p* < 0.01). These findings suggest that autonomic responses in complex interactive environments may reflect dynamic engagement processes rather than uniform stress activation, supporting multimodal cognitive load assessment and offering transferable insights for interface design and workload evaluation in demanding HCI contexts.

## 1. Introduction

Human–computer interaction (HCI) interfaces play a pivotal role in industrial applications, where they facilitate complex operations in sectors such as manufacturing, aviation, and healthcare. These interfaces often demand high cognitive resources from users, involving multitasking, rapid decision making, and processing dense visual information under time constraints, which can lead to elevated cognitive load and potential errors [1,2,3,4]. Cognitive load theory (CLT), proposed by Sweller, posits that cognitive load—the mental effort required to process information and execute tasks—comprises three types: intrinsic load (inherent task complexity), extraneous load (unnecessary burden from poor design), and germane load (beneficial effort for schema construction and learning) [5,6]. In the present study, we draw on CLT as a conceptual framework to distinguish between different types of cognitive demands (intrinsic load from task complexity, extraneous load from interface design, and germane load from learning-oriented reflection), rather than to rigidly classify each experimental manipulation into one of the three categories. This framework is crucial for understanding operational efficiency and safety in high-stakes environments, as excessive total load, limited by working memory capacity, can result in reduced performance, increased fatigue, and higher error rates [2,4,5,7]. Thus, adaptive HCI designs that minimize extraneous load while optimizing intrinsic and germane loads are essential to mitigate these burdens [1,2,7,8].

To effectively measure and manage cognitive load in HCI, researchers have employed both subjective and objective methods. Subjective tools, such as the NASA Task Load Index (NASA-TLX) and System Usability Scale (SUS), offer insights into perceived workload but are prone to biases like recall errors and individual variability [3,9,10,11]. For example, the NASA-TLX has been widely validated in HCI studies for its multidimensional assessment of workload, yet it relies on post-task self-reports that may not capture real-time fluctuations [12]. Objective physiological indicators, particularly heart rate variability (HRV), provide real-time, non-intrusive assessments, with reduced HRV (e.g., lower RMSSD or HF components) signaling heightened sympathetic activation and cognitive strain [2,13,14,15,16]. HRV has been correlated with cognitive performance across domains, demonstrating that high demands lead to decreased variability and impaired executive functions, such as in dual-task paradigms where HRV metrics predict error rates [2,13,14,15].

Despite the growing interest in physiological workload estimation, the existing literature has largely focused on domain-specific applications or isolated task dimensions, without providing a comprehensive analysis of methodologies that integrate both physical and cognitive workload across different contexts [2]. As noted in a recent systematic review [17], most studies rely on controlled laboratory tasks (e.g., N-back tests and driving simulations) with limited generalizability to real-world scenarios, highlighting the need for multimodal approaches in ecologically valid settings. The review further emphasizes the need for multimodal monitoring, standardized protocols, and adaptive models that can handle inter-individual variability—challenges that are particularly relevant to dynamic human–computer interaction (HCI) environments. Moreover, recent methodological work has demonstrated that games can serve as valid research instruments for studying cognitive and behavioral processes in complex, dynamic environments, provided that their design aligns with the target context [18,19].

**Figure 1:**
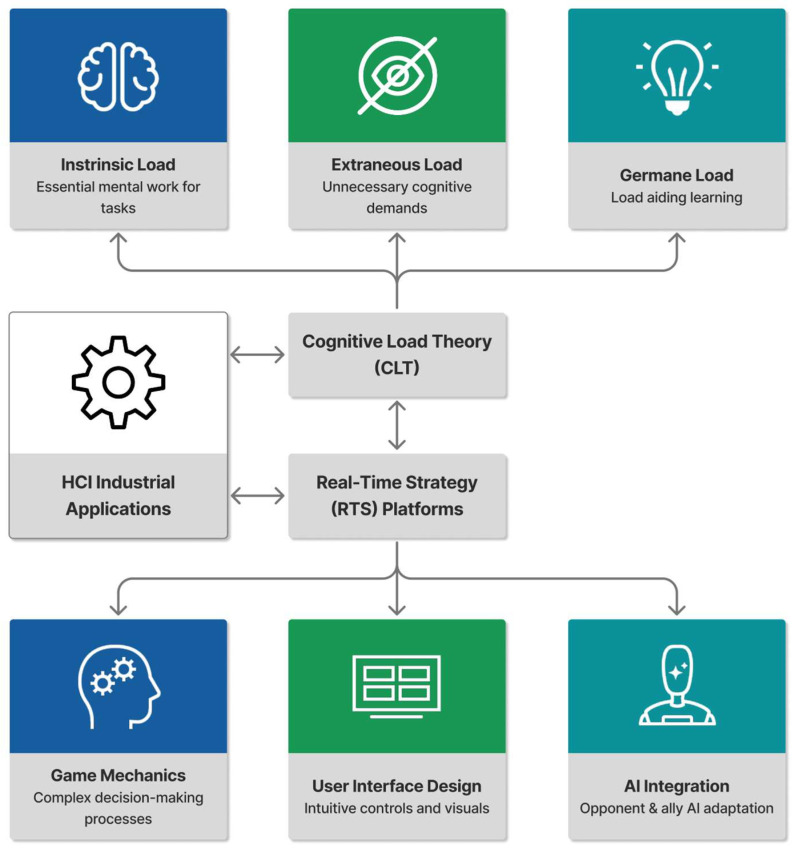
Conceptual framework bridging HCI industrial applications, cognitive load theory (CLT), and RTS platforms.

As illustrated in Figure 1, real-time strategy (RTS) games offer a unique platform to address precisely these methodological challenges. Unlike traditional laboratory tasks, RTS games inherently combine multiple cognitive demands—such as multitasking, visual complexity, and decision pressure—within an interactive, ecologically valid setting [7,17,18,19]. They require players to manage resources, monitor multiple information streams, and make rapid decisions under time constraints, closely mirroring the demands of industrial HCI applications like air traffic control, emergency response, and process monitoring [20,21].

Previous RTS research has largely focused on gameplay performance or AI behavior, with limited attention to the real-time physiological assessment of cognitive load [7,20,21,22,23,24]. Critically, existing studies often treat cognitive load as a unidimensional construct, overlooking the interplay between its intrinsic, extraneous, and germane components [2,17], and have rarely examined how HRV—a well-established indicator of autonomic arousal in controlled tasks [13,14,16,25]—responds to distinct cognitive load dimensions in complex interactive environments.

To address these gaps, the present study adopts a multimodal approach that integrates wearable HRV monitoring, subjective workload assessment (raw NASA-TLX), behavioral metrics, and qualitative interviews. By systematically manipulating three core dimensions of cognitive load—multitasking, visual complexity, and real-time decision pressure—within a controlled RTS environment, we aim to: (1) characterize the effects of each dimension on subjective, behavioral, and physiological responses; (2) examine the relationships among these modalities; and (3) evaluate the potential of a brief reflection intervention to support germane load. This study contributes to the literature by providing empirical evidence for multimodal cognitive load assessment in an ecologically valid HCI context, with implications for adaptive interface design in high-demand industrial applications.

The following hypotheses are proposed, with the expectation that multimodal measurements will not only test these causal effects but also reveal the interrelationships among physiological, subjective, and behavioral indicators of cognitive load:

**H1.**  *Higher levels of multitasking in RTS interfaces will lead to increased physiological load, increased subjective cognitive load and poorer behavior compared to lower levels*.

**H2.**  *Higher levels of visual complexity in RTS interfaces will lead to increased physiological load, increased subjective cognitive load and poorer behavior compared to lower levels*.

**H3.**  *Higher levels of real-time decision pressure in RTS interfaces will lead to increased physiological load, increased subjective cognitive load and poorer behavior compared to lower levels*.

## 2. Materials and Methods

**Figure 2:**
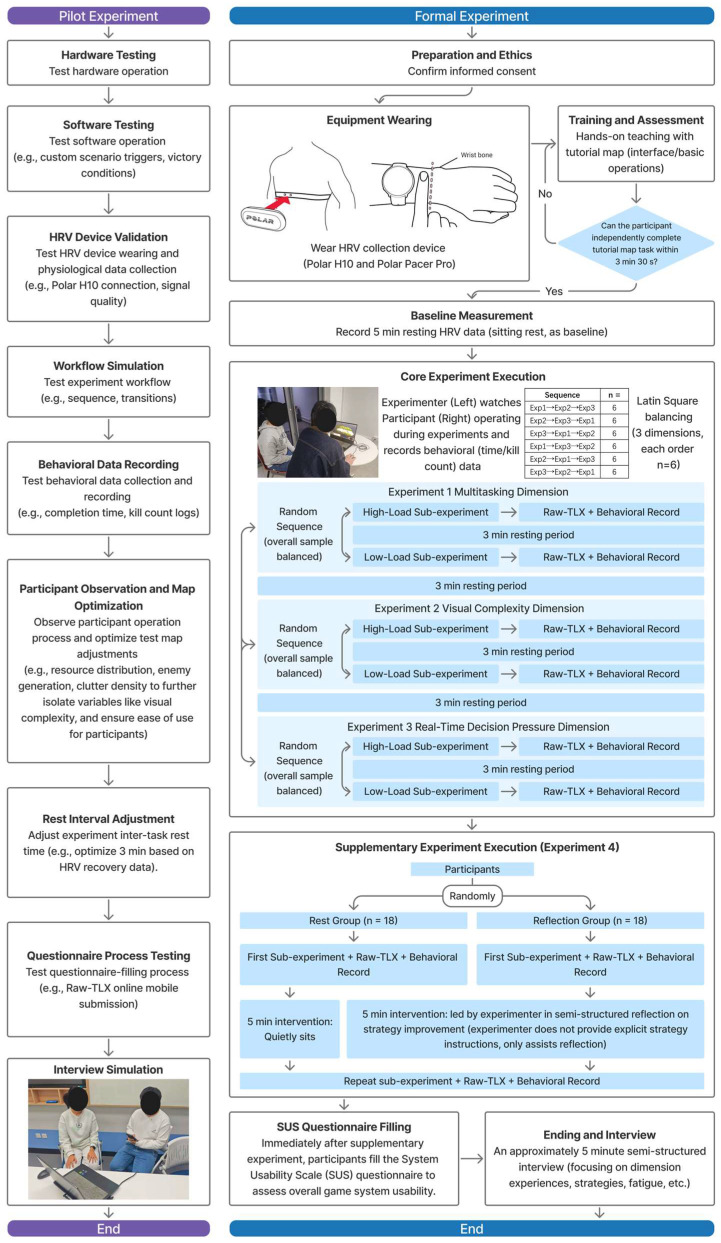
Overall experimental procedure.

Figure 2 presents an overview of the overall experimental procedure. The study consisted of a pilot phase to validate and optimize the protocols, followed by a formal experiment that included four phases: three core experiments (multitasking, visual complexity, and real-time decision pressure) and one supplementary experiment (germane load contrast). The details of the experimental setup, participant recruitment, and the protocol for each phase are described in the following subsections.

### 2.1. System and Experimental Environment

The experiment was conducted using *Age of Empires II: Definitive Edition* (Update162286; Microsoft, Redmond, WA, USA, 2019) as the primary platform, customized via the in-game Scenario Editor to create controlled scenarios for each dimension of cognitive load. All tasks were implemented in the Feudal Age setting to ensure consistency in game mechanics. Participants interacted with the game on standardized notebook computers (Intel Core i9 processor, 32 GB RAM, 2560 × 1600 resolution monitors) equipped with a keyboard and mouse. Heart rate variability (HRV) was measured using the Polar H10 chest strap sensor (Polar Electro Oy, Finland), with data collected and later analyzed offline using Kubios HRV Scientific software (version 4.0; Kubios Oy, Kuopio, Finland). This setup allowed for the non-invasive monitoring of autonomic nervous system activity, aligning with established protocols for cognitive load assessment in human–computer interaction (HCI) studies [2].

The Polar H10 chest strap (Polar Electro Oy, Kempele, Finland) was selected for HRV monitoring due to its lightweight, unobtrusive design, which minimized participant burden during the extended experimental session (approximately 90 min) and reduced the risk of sensor-related distractions or fatigue.

### 2.2. Participant Recruitment and Grouping

Participants were recruited through university forums, social media, and personal networks, targeting 36 novice players (aged 20–28 years) with minimal *Age of Empires II* game experience (<10 h lifetime) but allowing limited other real-time strategy (RTS) or multiplayer online battle arena (MOBA) exposure. An online screening questionnaire assessed demographics, gaming history, health conditions (e.g., excluding cardiac issues that could affect HRV), and availability. Informed consent emphasized voluntary participation and data anonymity [26].

Following recruitment, a total of 36 participants (21 males, 15 females; mean age = 22.97 years, SD = 2.22, range = 20–28 years) were enrolled. The age distribution showed a concentration in the early twenties, with 7 participants each at ages 21, 22, and 23, followed by 5 at age 26, 4 each at ages 20 and 25, and 1 each at ages 27 and 28. Health screening confirmed that 33 participants (91.67%) reported no health issues, while 3 (8.33%) reported other minor conditions not affecting study eligibility.

Participants’ gaming habits varied: 13 (36.11%) played games daily, 10 (27.78%) occasionally (a few times a month), 9 (25%) frequently (a few times a week), and 4 (11.11%) never played. In terms of game type exposure (multiple selections allowed), the most common were first-person shooters (FPSs, e.g., *CS:GO*; *n* = 17) and multiplayer online battle arenas (MOBAs, e.g., *League of Legends*; n = 17), followed by other types (*n* = 13), real-time strategy (RTS, e.g., *StarCraft*; *n* = 11), and role-playing games (RPGs, e.g., *The Legend of Zelda*; *n* = 10).

**Figure 3:**
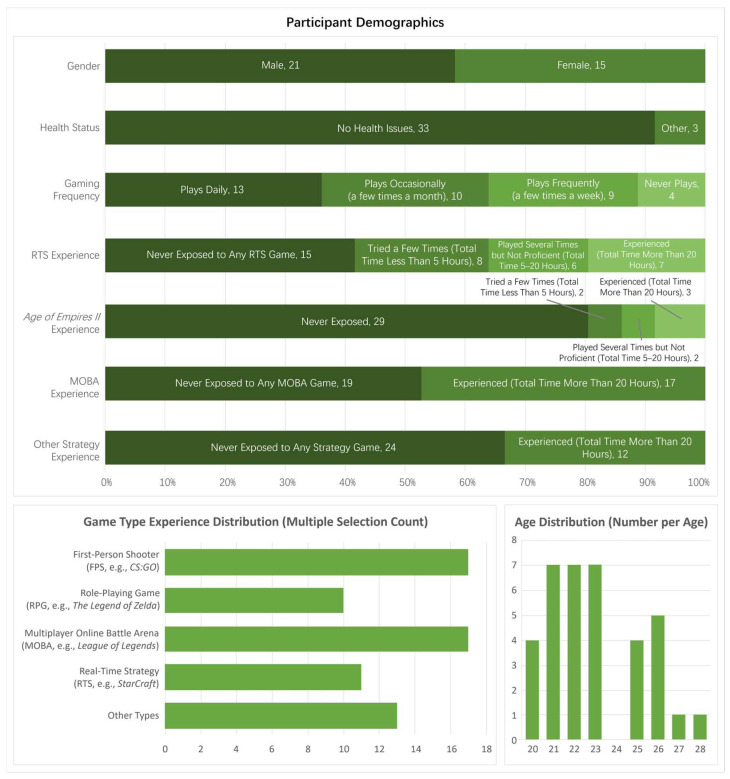
Demographic characteristics of participants.

Consistent with inclusion criteria, RTS experience was limited: 15 (41.67%) had never been exposed to any RTS game, 8 (22.22%) had tried a few times (total time < 5 h), 6 (16.67%) had played some RTS games but not proficiently (total time 5–20 h), and 7 (19.44%) were experienced (total time > 20 h). Specifically for *Age of Empires II*, 29 (80.56%) had never been exposed, 2 (5.56%) had tried a few times (<5 h), 2 (5.56%) had played several times (5–20 h), and 3 (8.33%) were experienced (>20 h), ensuring all had <10 h lifetime exposure. MOBA experience was split: 19 (52.78%) had never been exposed, and 17 (47.22%) were experienced (>20 h). For other strategy games, 24 (66.67%) had never been exposed, and 12 (33.33%) were experienced (>20 h). These demographics are summarized in Figure 3.

A within-subjects design was used for the core experiments (dimensions 1–3), with Latin Square counterbalancing to control order effects [27]. Sample size was determined via a priori power analysis using G*Power software (version 3.1; Heinrich-Heine-Universität Düsseldorf, Düsseldorf, Germany [28]). Given the within-subjects design and the use of a paired *t*-test (two-tailed), with an anticipated effect size (dz) of 0.5, an alpha error probability of 0.05, and a desired statistical power of 0.80, the required sample size was determined to be 34 participants. In the present study, a total of 36 participants were recruited to ensure adequate power.

For the supplementary experiment (germane load), a between-subjects design randomly assigned participants to rest (*n* = 18) or reflection (*n* = 18) groups using stratified randomization based on age and gender.

### 2.3. Experimental Procedure

The experimental procedure was divided into a pilot phase (*n* = 10) to validate and optimize protocols, followed by the formal phase (*n* = 36) for data collection. The overall workflow is illustrated in Figure 2, which details the sequence of hardware/software testing, participant training, core and supplementary experiments, and data recording.

#### 2.3.1. Pilot Experiment

Ten participants were recruited to test hardware stability (e.g., game platform), software functionality (e.g., custom triggers), HRV device validation (e.g., Polar H10 signal quality), workflow simulation (e.g., transitions), behavioral data logging (e.g., completion time and kill counts), rest interval adjustments (optimized to 3 min based on HRV recovery), questionnaire processes (e.g., raw-TLX mobile submission), and interview simulations. Participant observations led to map optimizations (e.g., resource distribution, enemy generation, and clutter density) to isolate variables and enhance usability. Issues were logged in Excel for iterative refinements, ensuring efficiency in the formal phase.

#### 2.3.2. Formal Experiment

**Figure 4:**
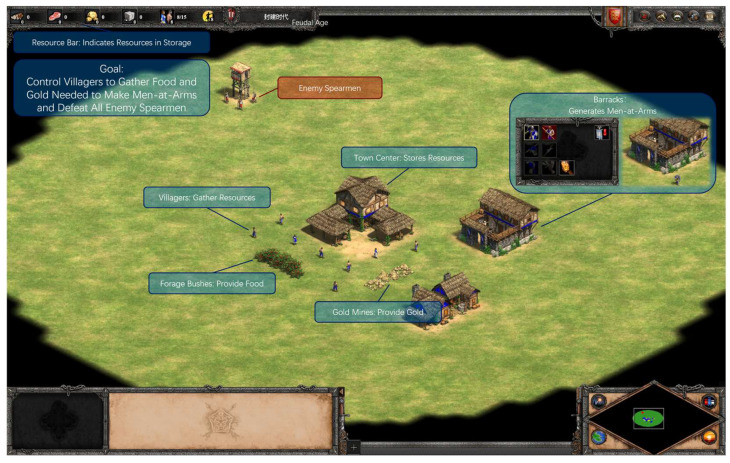
Tutorial scenario.

Following informed consent, participants wore the Polar H10 for HRV monitoring. All experiments were conducted in silence throughout the gameplay. The same experimenter conducted a hands-on tutorial using a custom map to train basic operations (see Figure 4), with independent completion required within 3 min 30 s (repeated if needed). A 5 min baseline HRV was recorded at rest.

The experiment comprised four phases, which were designed based on cognitive load theory (CLT) to isolate negative loads (intrinsic/extraneous) in the core phases and germane load in the supplementary phase [27,29].

Core experiments (dimensions 1–3) used a within-subjects design with Latin Square counterbalancing (6 orders, *n* = 6 each) and randomized high/low sub-experiment sequences per dimension (balanced overall). Each sub-experiment lasted 5–7 min, followed by 3 min rests and immediate raw-TLX completion via mobile.

The supplementary experiment (germane load) employed a between-subjects design (rest group *n* = 18; reflection group *n* = 18). It involved an initial sub-experiment and raw-TLX, a 5 min intervention (quiet rest or guided reflection on strategies), and a repeated sub-experiment and raw-TLX. Post-supplementary experiment, SUS was administered, followed by a semi-structured interview on experiences, strategies, and fatigue.

#### 2.3.3. Experiment 1: Multitasking Dimension

This dimension was set in the Feudal Age with full technologies unlocked. The town center buttons were disabled, and the barracks allowed only Men-at-Arms production. Participants started with 0 resources, wood piles scattered around the initial town center, and five villagers (no new villagers could be built). All elements were within the locked screen view.

**Figure 5:**
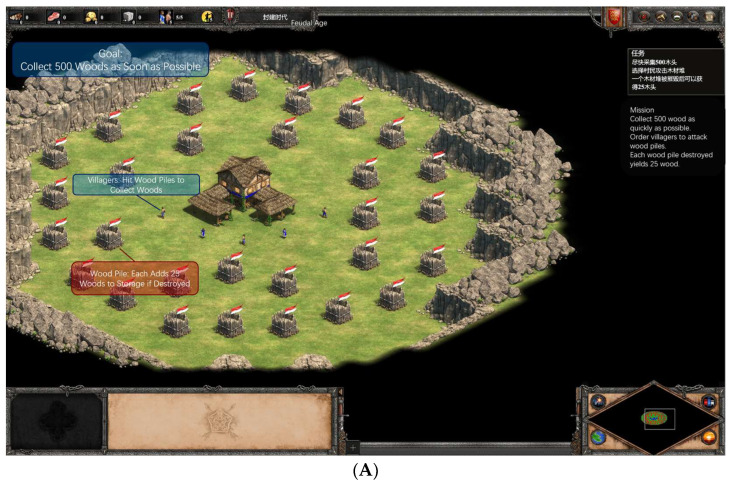

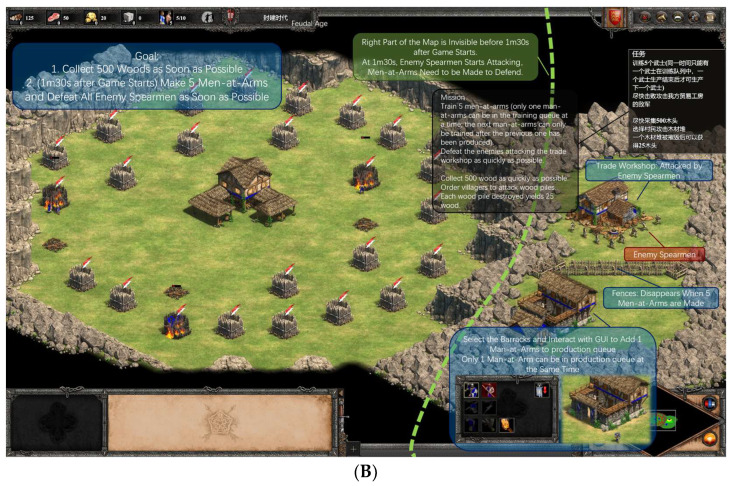
(**A**) Scenario for multitasking dimension (low load level). (**B**) Scenario for multitasking dimension (high load level).

- Low Load (Single-Thread): Participants controlled 5 villagers to collect 500 units of wood from scattered piles. No combat or additional threads. Victory triggered upon reaching the resource threshold, stopping the timer (see Figure 5A).
- High Load (Dual-Thread): Participants managed the low-load resource collection simultaneously. After 1.5 min (unbeknownst to players), the right screen revealed the player’s trade workshop under attack by 10 enemy spearmen, requiring Men-at-Arms production for defense. The barracks, fenced off below the workshop, unlocked after producing 5 Men-at-Arms (initial resources for one; subsequent resources granted per unit to prevent queuing all at once). Enemy spearmen followed default AI for seeking and attacking. Victory triggered upon resource threshold and defeating all enemies or all player units dead, stopping the timer (see Figure 5B).

Behavioral Performance Indicator: Completion time.

#### 2.3.4. Experiment 2: Visual Complexity Dimension

The map featured a linear navigation path where participants controlled a scout cavalry unit to reach a designated endpoint, with free screen view. There were no resources or combat; the focus was solely on visual processing.

**Figure 6:**
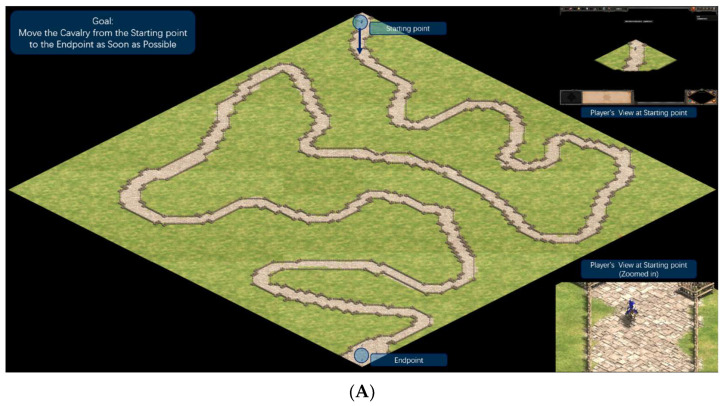

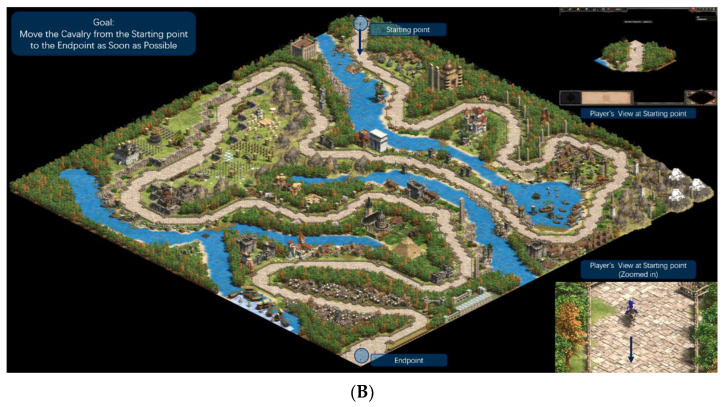
(**A**) Scenario for visual complexity dimension (low load level). A high-resolution version of this scenario is available in Supplementary Materials Figure S1. (**B**) Scenario for visual complexity dimension (high load level). A high-resolution version of this scenario is available in Supplementary Materials Figure S2.

- Low Load: Scene included only essential elements: road, collision-controlling barriers along edges, and blank grassy background, minimizing visual clutter for direct navigation. Victory triggered upon arrival, stopping the timer (see Figure 6A).
- High Load: Identical base elements (road and barriers) as low load, but added decorative landmarks (e.g., ruins, rocks, flags, extra trees, non-interactive buildings, AI units in combat) to increase visual density and attentional demands without altering the path. Victory triggered upon arrival, stopping the timer (see Figure 6B).

Behavioral Performance Indicator: Completion time.

#### 2.3.5. Experiment 3: Real-Time Decision Pressure Dimension

**Figure 7:**
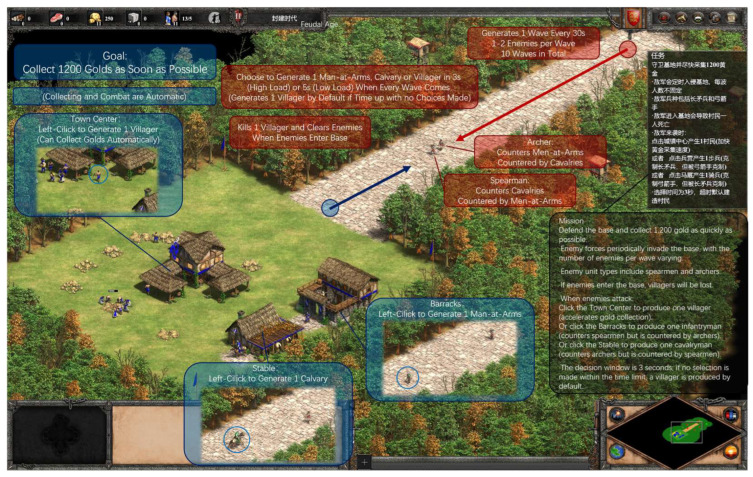
Scenario for real-time decision pressure dimension (low and high load level).

Participants started in the Feudal Age with a town center, barracks, stable, and initial villagers for gold mining. The custom map included gold mines and generated enemy waves at fixed intervals (every 30 s, 10 waves total, 1–2 enemies per wave: spearmen or archers). Each wave prompted a decision: click town center for 1 villager, barracks for 1 Man-at-Arms (counters spearmen), or stable for 1 cavalry (counters archers). The decisions were time-limited; timeout defaulted to 1 villager. Custom unit stats met experimental needs. If enemies reached the base, 1 villager was scripted to be lost. Collecting 1200 units of gold triggered automatic victory. All elements were within locked screen view; villagers auto-mined, soldiers auto-moved/attacked; players could only click buildings (see Figure 7).

- Low Load: 5 s decision window.
- High Load: 3 s decision window.

Behavioral Performance Indicator: Completion time.

#### 2.3.6. Experiment 4: Germane Load Contrast

**Figure 8:**
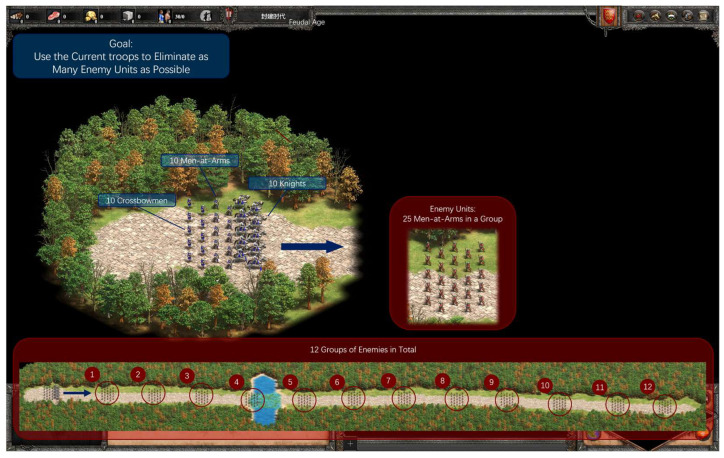
Scenario for germane load contrast. A high-resolution version of this scenario is available in Supplementary Materials Figure S3.

As a supplementary experiment, this experiment aimed to investigate the role and impact of germane load based on CLT. This between-subjects phase used a linear road map with 12 groups of 25 enemy Men-at-Arms distributed along a straight path from left to right, with free screen view. Participants controlled 10 Men-at-Arms, 10 crossbowmen, and 10 knights to eliminate as many enemies as possible before troop annihilation. There were identical maps for both trials to assess learning effects (see Figure 8).

- Procedure: Initial trial and raw-TLX. Five min intervention: rest group (quiet sitting); reflection group (facilitator prompted strategy reflection without suggestions). Repeated trial and raw-TLX.

Behavioral Performance Indicator: Kill count (pre–post comparison).

### 2.4. Measurement Metrics

Cognitive load was assessed using a multimodal approach, combining physiological, behavioral, and subjective measures to capture real-time and perceived workload [2].

- Physiological Metrics (HRV): Heart rate variability was analyzed using Kubios software (version 4.0). Indices in Table 1 were computed to evaluate autonomic nervous system balance [30].
- Behavioral Metrics: Completion time (in seconds) for Experiments 1–3 (multitasking, visual complexity, real-time decision pressure), reflecting task efficiency; kill count (total enemies eliminated) for Experiment 4 (germane load), assessing performance improvement.
- Subjective Metrics: raw NASA-TLX (as raw-TLX, 6 dimensions: mental demand, physical demand, temporal demand, performance, effort, frustration; simply summed overall score) administered post-sub-experiment to gauge perceived workload [12].
- Usability Metrics: System Usability Scale (SUS) administered at the end to evaluate overall game system usability [10].

### 2.5. Data Preprocessing and Signal Quality Control

Raw HRV data were preprocessed using Microsoft Excel to ensure signal integrity and minimize artifacts. Initially, RR intervals (the time between consecutive R-peaks in the ECG signal) were extracted from the Polar H10 recordings. Signal quality control involved identifying potential artifacts through a multi-criteria approach: RR intervals shorter than 400 ms or longer than 1200 ms (adjusted based on individual variability to account for resting heart rates typically between 50–150 bpm) were flagged as suspicious. Additionally, intervals exhibiting a relative deviation exceeding 20% from the session’s mean RR, combined with an adjacent change rate over 20%, were also marked as potential artifacts [15,20,31,32]. These 20% values refer to detection thresholds, not the proportion of corrected intervals.

For flagged artifacts, manual inspection and correction were applied: If two adjacent RR intervals appeared erroneously merged (e.g., due to missed beats), they were replaced with the linear average of the preceding and following valid intervals. If a single RR interval seemed split into two (e.g., due to extra beats), the segments were merged into one corrected value. Other significant deviations from normative ranges were interpolated using the linear average of neighboring valid RR intervals. This manual approach allowed us to maintain rigorous quality control across the dataset. Importantly, it enabled per-participant adjustment of detection criteria: For each participant, flagged intervals were examined against their own baseline RR distribution (derived from the 5 min resting period) and ECG waveform morphology. Intervals that fell within the expected physiological range for that individual—for example, a baseline RR of 1200 ms in an athletic participant—were retained as normal, while intervals that were clearly artifactual (e.g., motion-induced spikes) were corrected using linear interpolation. This ensured that genuine physiological variability was preserved while true artifacts were reliably removed. This ensured artifact-free data for the subsequent Kubios analysis, with less than 5% of intervals requiring correction across sessions [15,30,31,32].

### 2.6. Statistical Analysis

All statistical analyses were conducted using Python (version 3.12) with libraries such as SciPy and Statsmodels for hypothesis testing. Normality was assessed for each metric using the Shapiro–Wilk test (α = 0.05).

For Experiments 1–3 (within-subjects design: multitasking, visual complexity, real-time decision pressure), paired comparisons between high- and low-load conditions were performed. For the HRV metrics, the comparisons were based on the relative change rates from baseline for high (H) and low (L) conditions respectively. For the subjective and behavioral data, the comparisons used the raw scores and metrics from H and L conditions. The metrics meeting normality assumptions underwent paired *t*-tests, while the non-normal data were analyzed via Wilcoxon signed-rank tests. The effect sizes were calculated as Cohen’s d for *t*-tests and rank-biserial correlation (r) for Wilcoxon tests.

For Experiment 4 (between-subjects design: germane load), independent comparisons between the rest and reflection groups (pre- and post-intervention) were conducted. For the HRV metrics, the comparisons were based on the relative change rates from pre to post for the rest and reflection groups. For the subjective data, the comparisons used the change values in scores from pre to post for the rest and reflection groups. For the behavioral data, the comparisons used the change values in enemy units killed from pre to post for the rest and reflection groups. The normal data were tested using independent samples *t*-tests, and the non-normal data via Mann–Whitney U tests. The effect sizes included Cohen’s d for *t*-tests and rank-biserial correlation (r) for U tests.

Additionally, paired correlation analyses (Pearson or Spearman, based on normality) were conducted to examine the relationships among the HRV metrics, subjective raw-TLX scores, and behavioral indicators (completion time, kill count). For Experiment 1–4, HRV correlations were based on relative change rates from baseline, while subjective and behavioral data correlations were based on raw scores. These analyses aimed to (1) validate the sensitivity of physiological measures to perceived workload, (2) explore the coupling between subjective experience and objective performance, and (3) provide supplementary evidence for interpreting between-group findings.

All tests were two-tailed with significance at α = 0.05, and the results were adjusted for multiple comparisons using Bonferroni correction where applicable.

### 2.7. Interview Data Analysis Method

To complement the quantitative findings, the semi-structured interview data were subjected to an AI-assisted thematic analysis adapted from Braun and Clarke [33]. Given the exploratory and supplementary nature of the qualitative component [34,35], the procedure emphasized structured theme identification while maintaining human oversight. Such AI-assisted methods have been shown to streamline initial coding and theme identification in qualitative research, particularly when human oversight is integrated to address potential biases and ensure contextual accuracy [36,37].

The dataset included 54 interview segments: 18 from the reflection phase in Experiment 4 and 36 from the final interviews. Anonymized transcripts were processed using Deepseek-V3.2 to assist in generating preliminary codes and candidate themes based on a structured prompt. The AI outputs were treated as initial suggestions rather than final analytical decisions.

All themes and representative quotes were reviewed and refined by the authors to ensure contextual appropriateness and conceptual consistency. Manual verification focused on cross-checking theme boundaries, merging overlapping categories, and removing AI-generated misclassifications. The finalized themes (3–4 per interview type) were then used for illustrative triangulation with quantitative measures (e.g., HRV and raw-TLX scores) in the discussion section.

To assess the reliability of the thematic frameworks, 36 coding units were randomly selected from the reflection interviews (3 themes), covering all 18 participants, and 52 units were randomly selected from the final interviews (4 themes), covering all 36 participants. Two independent coders—both participants in the experiment—were trained on the respective thematic definitions and independently classified each unit. For the reflection interviews, the inter-coder agreement was 75% (Cohen’s κ = 0.58, *p* < 0.05), indicating moderate agreement. For the final interviews, the agreement was 98% (κ = 0.92, *p* < 0.05), indicating almost perfect agreement. These results support the reliability of the thematic structures, with the slightly lower agreement for the reflection interviews reflecting the thematic proximity of the three themes, which occasionally co-occur in the same segment, making classification more challenging for coders without specialized training in qualitative analysis.

## 3. Results

### 3.1. Multitasking Dimension

**Figure 9:**
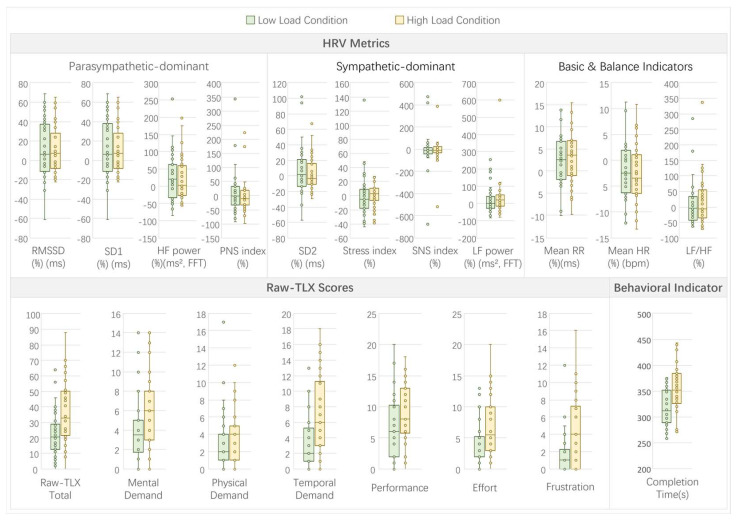
Results indicators under high-load and low-load conditions in Experiment 1.

In Experiment 1, which examined the effects of multitasking load, comparisons between high-load (dual-thread) and low-load (single-thread) conditions revealed distinct patterns across HRV, subjective, and behavioral metrics (see Figure 9 for a visual summary of indicators under high- and low-load conditions).

For HRV metrics, no significant differences were observed between the high- and low-load conditions (all *p* > 0.05; see Table A1). However, trends suggested a slight increase in sympathetic dominance under high load, such as a marginally higher stress index (high: 2.35% vs. low: −5.32%, W = 286, *p* = 0.4697, r = −0.12) and SNS index (high: −11.82% vs. low: −8.83%, W = 308, *p* = 0.7037, r = −0.07), though these were not statistically significant or marginally significant.

Subjective workload, as measured by raw-TLX, showed pronounced effects of multitasking load. Significant increases were found in mental demand (high: 6 vs. low: 3.5, W = 23.5, *p* < 0.001, r = −0.81), physical demand (high: 4 vs. low: 2, W = 52, *p* < 0.001, r = −0.74), temporal demand (high: 6 vs. low: 2, W = 31, *p* < 0.001, r = −0.79), performance (high: 8 vs. low: 6, W = 134.5, *p* = 0.0089, r = −0.52), effort (high: 5.5 vs. low: 2, W = 54.5, *p* < 0.001, r = −0.73), frustration (high: 4 vs. low: 1, W = 24.5, *p* < 0.001, r = −0.81), and total raw-TLX (high: 36.47 ± 20.19 vs. low: 22.50 ± 14.65, t(35) = 5.11, *p* < 0.001, d = 0.85). No marginally significant differences were noted in subjective metrics.

The behavioral indicator, completion time, was significantly longer under high load (high: 354.92 ± 50.70 s vs. low: 317.17 ± 37.26 s, t(35) = 4.77, *p* < 0.001, d = 0.80), indicating reduced task efficiency with increased multitasking demands.

**Figure A1:**
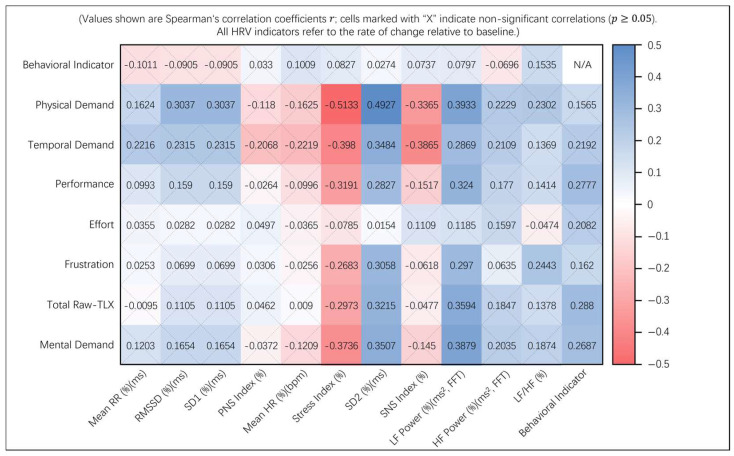
Heatmap of correlations among HRV, subjective ratings, and behavioral indicators in Experiment 1.

Correlations between the HRV parameters and subjective/behavioral measures revealed several significant associations (*p* < 0.05; see Figure A1). Parasympathetic-dominant metrics like RMSSD and SD1 positively correlated with mental demand (r = 0.3037, *p* = 0.0095). Sympathetic indicators showed negative correlations with multiple TLX dimensions, such as stress index with mental demand (r = −0.5133, *p* < 0.001), physical demand (r = −0.398, *p* < 0.001), and total raw-TLX (r = −0.3736, *p* = 0.0012). Long-term variability (SD2) and low-frequency power (LF power) positively correlated with several TLX scores, including mental demand (SD2: r = 0.4927, *p* < 0.001; LF power: r = 0.3933, *p* = 0.0006). Behavioral completion time positively correlated with temporal demand (r = 0.2777, *p* = 0.0182), frustration (r = 0.288, *p* = 0.0142), and total raw-TLX (r = 0.2687, *p* = 0.0225). These correlations suggest that physiological responses align with perceived workload under multitasking.

Full statistical results and correlation matrices for Experiment 1 are provided in Appendix A (Table A1 and Figure A1).

### 3.2. Visual Complexity Dimension

**Figure 10:**
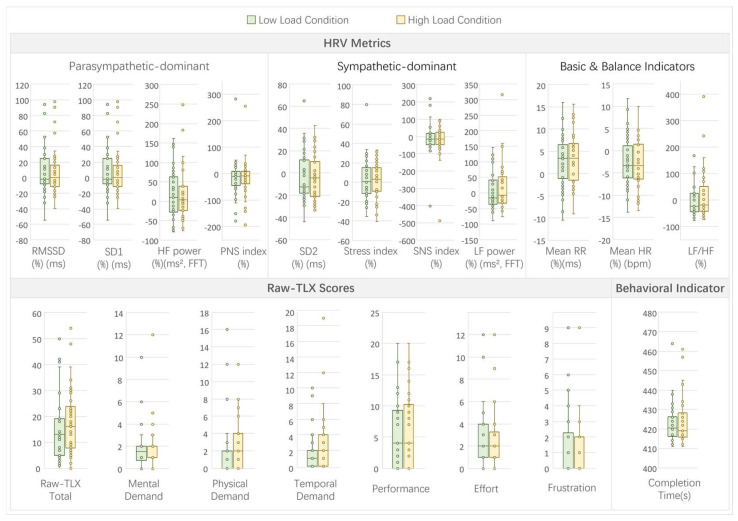
Results indicators under high-load and low-load conditions in Experiment 2.

In Experiment 2, which examined the effects of visual complexity load, comparisons between high-load and low-load conditions revealed limited patterns across HRV, subjective, and behavioral metrics (see Figure 10 for a visual summary of indicators under high- and low-load conditions).

For the HRV metrics, no significant differences were observed between the high- and low-load conditions (all *p* > 0.05; see Table A2). The trends indicated minor variations, such as a slight increase in sympathetic dominance under high load, including stress index (high: 3.23% vs. low: 0.68%, W = 294, *p* = 0.5401, r = −0.07) and SNS index (high: −15.34% vs. low: −13.63%, W = 267, *p* = 0.2998, r = −0.12), but these were not statistically significant or marginally significant.

Subjective workload, as measured by raw-TLX, showed the selective effects of visual complexity. A significant increase was found only in temporal demand (high: 2 vs. low: 1, W = 20, *p* = 0.043, r = −0.24). No significant differences were noted in mental demand (high: 2 vs. low: 1.5, W = 61, *p* = 0.7131, r = −0.04), physical demand (high: 2 vs. low: 2, W = 72, *p* = 0.552, r = −0.07), performance (high: 4 vs. low: 4, W = 64, *p* = 0.5679, r = −0.07), effort (high: 2 vs. low: 2, W = 148, *p* = 0.6896, r = −0.05), frustration (high: 0 vs. low: 0, W = 32, *p* = 0.1779, r = −0.16), or total raw-TLX (high: 16 vs. low: 13, W = 228, *p* = 0.6875, r = −0.05). No marginally significant differences were noted in subjective metrics.

The behavioral indicator, completion time, showed no significant difference between the conditions (high: 419 s vs. low: 420.5 s, W = 251, *p* = 0.5974, r = −0.06), suggesting that visual complexity did not substantially impact navigation efficiency.

**Figure A2:**
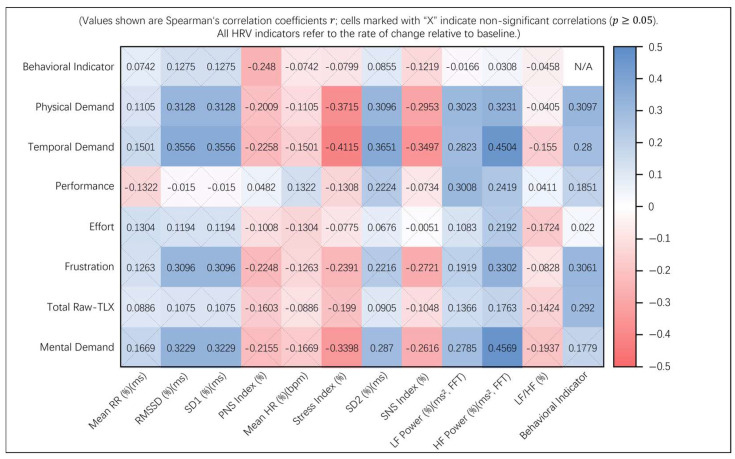
Heatmap of correlations among HRV, subjective ratings, and behavioral indicators in Experiment 2.

The correlations between the HRV parameters and subjective/behavioral measures revealed several significant associations (*p* < 0.05; see Figure A2). Parasympathetic-dominant metrics like RMSSD and SD1 positively correlated with mental demand (r = 0.3128, *p* = 0.0075), physical demand (r = 0.3556, *p* = 0.0022), effort (r = 0.3096, *p* = 0.0081), and total raw-TLX (r = 0.3229, *p* = 0.0057). Sympathetic indicators showed negative correlations with multiple TLX dimensions, such as stress index with mental demand (r = −0.3715, *p* = 0.0013), physical demand (r = −0.4115, *p* = 0.0003), effort (r = −0.2391, *p* = 0.0431), and total raw-TLX (r = −0.3398, *p* = 0.0035). The long-term variability (SD2) and low-frequency power (LF power) positively correlated with several TLX scores, including mental demand (SD2: r = 0.3096, *p* = 0.0081; LF power: r = 0.3023, *p* = 0.0099) and total raw-TLX (SD2: r = 0.287, *p* = 0.0145; LF power: r = 0.2785, *p* = 0.0178). High-frequency power (HF power) positively correlated with physical demand (r = 0.4504, *p* = 0.0001), temporal demand (r = 0.2419, *p* = 0.0406), effort (r = 0.3302, *p* = 0.0046), and total raw-TLX (r = 0.4569, *p* = 0.0001). Behavioral completion time positively correlated with mental demand (r = 0.3097, *p* = 0.0081), physical demand (r = 0.28, *p* = 0.0172), effort (r = 0.3061, *p* = 0.0089), and frustration (r = 0.292, *p* = 0.0128). These correlations indicate that physiological responses were linked to perceived workload, despite limited direct differences in visual complexity conditions.

Full statistical results and correlation matrices for Experiment 2 are provided in Appendix A (Table A2 and Figure A2).

### 3.3. Real-Time Decision Pressure Dimension

**Figure 11:**
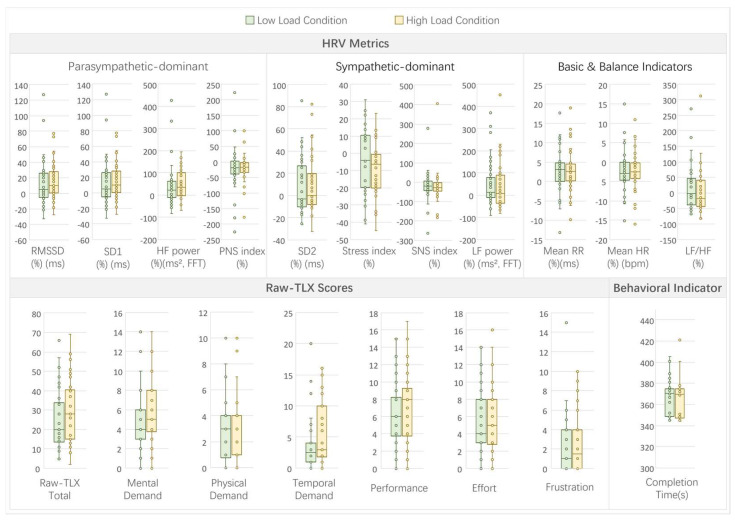
Results indicators under high-load and low-load conditions in Experiment 3.

In Experiment 3, which examined the effects of real-time decision pressure load, comparisons between high-load and low-load conditions revealed moderate patterns across the HRV, subjective, and behavioral metrics (see Figure 11 for a visual summary of indicators under high- and low-load conditions).

For the HRV metrics, no significant differences were observed between the high- and low-load conditions (all *p* > 0.05; see Table A3). However, the stress index showed a marginally significant decrease under high load (high: −8.75 ± 15.81% vs. low: −3.69 ± 18.29%, t(35) = −1.86, *p* = 0.0715, d = −0.31), suggesting a modest shift in autonomic balance under high decision pressure. This direction deviates from traditional stress-based models and should therefore be interpreted cautiously. 

The subjective workload, as measured by raw-TLX, showed notable effects of decision pressure. Significant increases were found in mental demand (high: 5 vs. low: 4, W = 43, *p* = 0.0019, r = 0.94), physical demand (high: 4 vs. low: 3, W = 61, *p* = 0.0281, r = 0.91), temporal demand (high: 3 vs. low: 2.5, W = 88, *p* = 0.0254, r = 0.87), and total raw-TLX (high: 28 vs. low: 20, W = 142.5, *p* = 0.008, r = 0.79). Frustration showed a marginally significant increase (high: 1.5 vs. low: 1, W = 54, *p* = 0.0538, r = 0.92). No significant differences were noted in performance (high: 6.92 ± 4.44 vs. low: 6.11 ± 4.00, t(35) = 1.45, *p* = 0.1553, d = 0.24) or effort (high: 5 vs. low: 4, W = 162, *p* = 0.5105, r = 0.76).

The behavioral indicator, completion time, showed no significant difference between the conditions (high: 369.5 s vs. low: 370.5 s, W = 272, *p* = 0.6625, r = 0.59), indicating that decision pressure did not substantially impact task completion efficiency.

**Figure A3:**
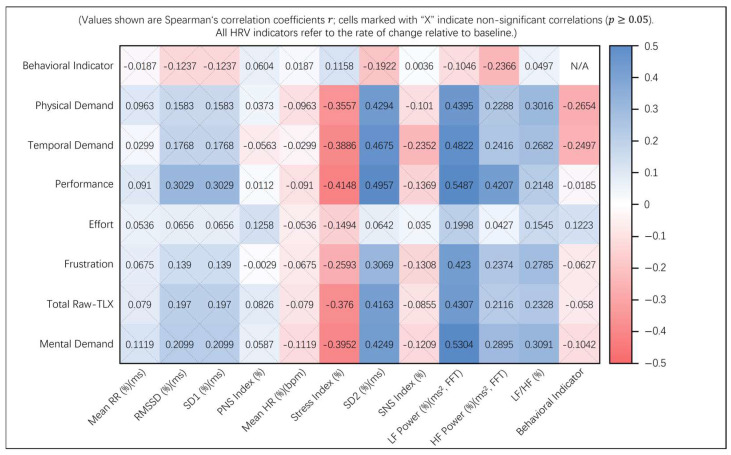
Heatmap of correlations among HRV, subjective ratings, and behavioral indicators in Experiment 3.

The correlations between the HRV parameters and subjective/behavioral measures revealed several significant associations (*p* < 0.05; see Figure A3). Parasympathetic-dominant metrics like RMSSD and SD1 positively correlated with temporal demand (r = 0.3029, *p* = 0.0097). Sympathetic indicators showed negative correlations with multiple TLX dimensions, such as stress index with mental demand (r = −0.3557, *p* = 0.0022), physical demand (r = −0.3886, *p* = 0.0007), temporal demand (r = −0.4148, *p* = 0.0003), effort (r = −0.2593, *p* = 0.0278), frustration (r = −0.376, *p* = 0.0011), and total raw-TLX (r = −0.3952, *p* = 0.0006). Long-term variability (SD2) and low-frequency power (LF power) positively correlated with several TLX scores, including mental demand (SD2: r = 0.4294, *p* = 0.0002; LF power: r = 0.4395, *p* = 0.0001) and total raw-TLX (SD2: r = 0.4249, *p* = 0.0002; LF power: r = 0.5304, *p* < 0.001). High-frequency power (HF power) positively correlated with physical demand (r = 0.2416, *p* = 0.0409), temporal demand (r = 0.4207, *p* = 0.0002), effort (r = 0.2374, *p* = 0.0447), and total raw-TLX (r = 0.2895, *p* = 0.0136). The LF/HF ratio positively correlated with mental demand (r = 0.3016, *p* = 0.01), physical demand (r = 0.2682, *p* = 0.0227), effort (r = 0.2785, *p* = 0.0178), frustration (r = 0.2328, *p* = 0.049), and total raw-TLX (r = 0.3091, *p* = 0.0082). Behavioral completion time negatively correlated with mental demand (r = −0.2654, *p* = 0.0243) and physical demand (r = −0.2497, *p* = 0.0344). These correlations suggest that physiological responses were associated with perceived workload under decision pressure conditions.

Full statistical results and correlation matrices for Experiment 3 are provided in Appendix A (Table A3 and Figure A3).

### 3.4. Germane Load Contrast

**Figure 12:**
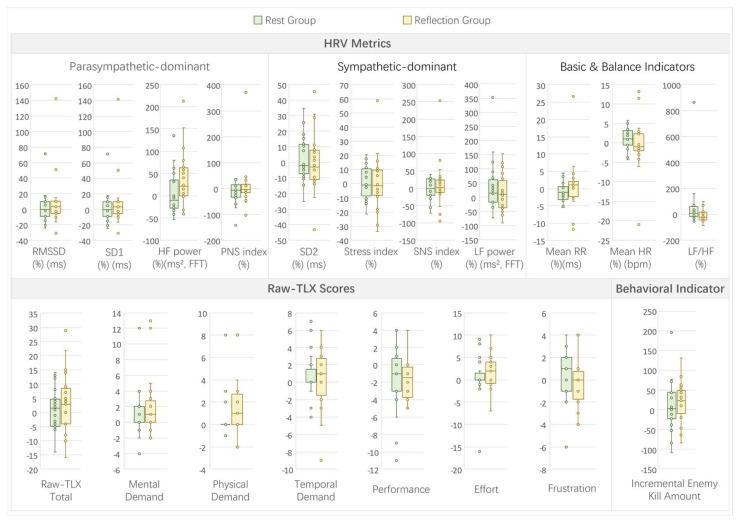
Pre-to-post task metric change ratio or amount in Experiment 4.

In Experiment 4, which examined the effects of germane load intervention (rest vs. reflection groups), the comparisons between pre- and post-intervention changes revealed minimal patterns across HRV, subjective, and behavioral metrics (see Figure 12 for a visual summary of pre-to-post task metric change ratios or amounts).

For the HRV metrics, no significant differences were observed between the rest and reflection groups (all *p* > 0.05; see Table A4). However, the LF/HF ratio showed a marginally significant difference, with a greater decrease in the reflection group (reflection: −21.77% vs. rest: 7.37%, U = 106, Z = −1.77, *p* = 0.079, r = 0.30), suggesting potential sympathovagal balance improvement through reflection.

The subjective workload, as measured by raw-TLX change values, showed no significant differences between the groups. This included mental demand (reflection: 1 vs. rest: 0, U = 191, Z = 0.92, *p* = 0.362, r = 0.15), physical demand (reflection: 1 vs. rest: 0, U = 193.5, Z = 1.00, *p* = 0.293, r = 0.17), temporal demand (reflection: 1 vs. rest: 0, U = 182, Z = 0.63, *p* = 0.531, r = 0.11), performance (reflection: −1.78 ± 2.26 vs. rest: −1.61 ± 4.05, t(34) = −0.15, *p* = 0.88, d = −0.05), effort (reflection: 2 vs. rest: 0, U = 185.5, Z = 0.74, *p* = 0.457, r = 0.12), frustration (reflection: 0 vs. rest: 1, U = 126, Z = −1.14, *p* = 0.257, r = 0.19), and total raw-TLX (reflection: 3.44 ± 11.59 vs. rest: 1.28 ± 7.47, t(34) = 0.67, *p* = 0.509, d = 0.22). No marginally significant differences were noted in the subjective metrics beyond the noted HRV.

The behavioral indicator, change in enemy kill amount, showed no significant difference between the groups (reflection: 20.78 ± 52.56 vs. rest: 13.06 ± 70.12, t(34) = 0.37, *p* = 0.711, d = 0.12), indicating that the reflection intervention did not substantially enhance performance improvement.

**Figure A4:**
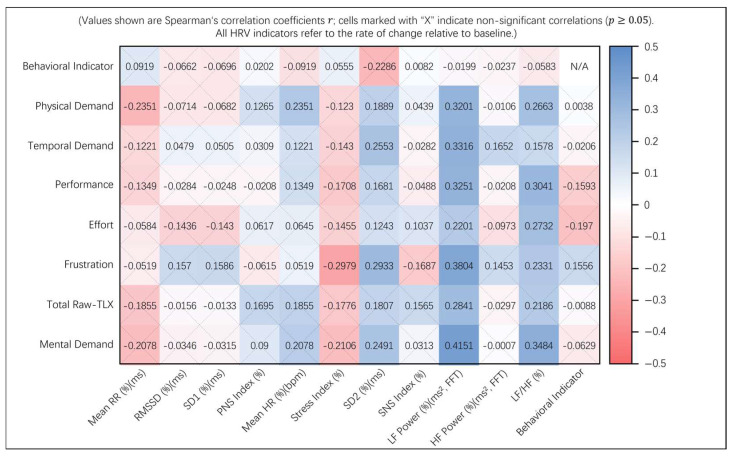
Heatmap of correlations among HRV, subjective ratings, and behavioral indicators in Experiment 4.

The correlations between HRV parameters and subjective/behavioral measures revealed several significant associations (*p* < 0.05; see Figure A4). Basic indicators like mean RR negatively correlated with mental demand (r = −0.2351, *p* = 0.0469), while mean HR positively correlated with mental demand (r = 0.2351, *p* = 0.0469). Sympathetic indicators showed negative correlations, such as stress index with effort (r = −0.2979, *p* = 0.011). Long-term variability (SD2) positively correlated with physical demand (r = 0.2553, *p* = 0.0304), effort (r = 0.2933, *p* = 0.0124), and total raw-TLX (r = 0.2491, *p* = 0.0349). Low-frequency power (LF power) positively correlated with mental demand (r = 0.3201, *p* = 0.0061), physical demand (r = 0.3316, *p* = 0.0044), temporal demand (r = 0.3251, *p* = 0.0053), effort (r = 0.3804, *p* = 0.001), frustration (r = 0.2841, *p* = 0.0156), and total raw-TLX (r = 0.4151, *p* = 0.0003). The LF/HF ratio positively correlated with mental demand (r = 0.2663, *p* = 0.0238), temporal demand (r = 0.3041, *p* = 0.0094), performance (r = 0.2732, *p* = 0.0202), effort (r = 0.2331, *p* = 0.0488), and total raw-TLX (r = 0.3484, *p* = 0.0027). These correlations suggest that physiological changes were linked to perceived workload shifts, despite the limited group differences in germane load conditions.

Full statistical results and correlation matrices for Experiment 4 are provided in Appendix A (Table A4 and Figure A4).

### 3.5. Cross-Experiment Comparison of HRV–Subjective–Behavioral Associations

To systematically compare the patterns of association across the four experiments, we examined the correlations between the HRV metrics (relative change from baseline), subjective ratings (raw NASA-TLX scores), and behavioral performance (completion time in Experiments 1–3; kill count in Experiment 4). Table 2 summarizes the directionality and significance of these relationships, highlighting both cross-experiment consistencies and task-specific dissociations.

All correlations reported in this section are exploratory. Given the large number of correlation tests across experiments, no formal multiple comparison correction was applied to the full set of analyses. Instead, we focus on cross-experiment consistency patterns (e.g., whether a given HRV metric shows the same directional association with subjective load across multiple experiments) as the primary basis for interpretation. Individual *p*-values are reported for transparency but should be interpreted as hypothesis-generating rather than confirmatory.

Key Observations:

- Sympathetic-related indices (particularly the stress index and SNS index) showed consistent negative correlations with subjective workload across experiments. That is, higher perceived demand was associated with lower sympathetic-related HRV indicators. This pattern appears to deviate from the classical workload model, which predicts increasing cognitive demand should elevate sympathetic activation.
  Several mechanisms may account for this divergence. First, under cognitively engaging tasks, increased attentional focus may enhance regulatory vagal control rather than purely activating sympathetic arousal. According to the neurovisceral integration framework, adaptive cognitive engagement involves coordinated prefrontal–autonomic regulation, which may manifest as flexible modulation rather than simple sympathetic dominance.
  Second, the present tasks required strategic adaptation rather than sustained stress exposure. Participants may have shifted toward controlled goal-directed regulation, reducing stress-related sympathetic signatures even when subjective workload remained high.
  Third, individual differences in coping strategies may contribute to inverse correlations at the individual level despite minimal group-level HRV shifts.
  Importantly, these findings should not be interpreted as evidence that cognitive load reduces sympathetic activation per se. Rather, one possible interpretation is that autonomic responses during complex interactive tasks could be influenced by engagement-related regulatory dynamics in addition to stress-driven arousal. However, given the absence of significant group-level HRV differences, this interpretation remains speculative and requires further validation.
- Global autonomic indices (LF power, SD2, LF/HF) exhibited robust positive correlations with subjective load in all four experiments, underscoring their value as stable markers of overall autonomic arousal during cognitive tasks.
- Parasympathetic indices displayed task dependent associations: RMSSD/SD1 were positively related to subjective load only in multitasking and visual complexity; HF power correlated positively with subjective load in visual complexity and decision pressure, and negatively with completion time in the decision pressure task—suggesting that vagal activation can both reflect perceived demand and facilitate performance under certain conditions.
- Subjective–behavioral relationships reversed across tasks: in multitasking and visual complexity, a higher subjective load predicted longer completion times (positive correlations), whereas under time pressure (Exp3), higher mental and physical demand were associated with faster completion, consistent with strategic adaptation.
- Physiological–behavioral links were rare but informative: a higher PNS index predicted faster navigation in visual complexity (Exp2), and a higher HF power predicted faster decision making under time pressure (Exp3), indicating that parasympathetic activity may support efficient performance in specific contexts.

These cross-experimental comparisons reveal both stable associations (e.g., LF power, SD2) and task-specific dissociations (e.g., reversed subjective–behavioral link in Exp3), which are discussed further in Section 4.3 and the respective task-specific subsections.

To assess the robustness of the key findings against Type I error, we performed a sensitivity analysis borrowing the Bonferroni principle on the 26 key correlation pairs derived from the Key Observations (see Table A5). Rather than applying a formal correction to the full set of exploratory correlations, we adopted a conservative threshold (α = 0.05/26 ≈ 0.0019) to examine whether the main patterns would survive under the most stringent conditions.

After applying this conservative threshold:

- Stress index showed significant negative correlations with subjective load in Experiments 1 and 3 (*p* < 0.0019 in both).
- LF power remained significantly correlated with total TLX in Experiments 1, 3, and 4 (*p* < 0.0019 in all three).
- SD2 remained significantly correlated with total TLX in Experiment 3 (*p* < 0.0019).
- HF power remained significantly correlated with total TLX in Experiment 2 (*p* < 0.0019).

The remaining core patterns—including the negative correlations of stress index in Experiments 2 and 4, and the positive correlations of SD2 in other experiments, as well as LF/HF with total TLX—showed consistent directional trends across all experiments where they were tested, though they did not meet the conservative threshold.

The subjective–behavioral and physiological–behavioral links exhibited the predicted directional patterns in specific experiments but did not survive this conservative test, consistent with their exploratory nature.

These results indicate that the core conclusions—particularly the cross-experiment consistency of sympathetic-related and global autonomic indices—are not driven by Type I error, even under the most stringent statistical criteria.

### 3.6. System Usability Scale (SUS) Results

To evaluate the overall perceived usability of the game system, participants completed the System Usability Scale (SUS) at the end of the experiment. The SUS is a 10-item questionnaire with alternating positive and negative items, each scored on a 5-point Likert scale (1 = Strongly Disagree, and 5 = Strongly Agree). The total SUS score ranges from 0 to 100, with higher scores indicating better perceived usability [10,11].

**Figure 13:**
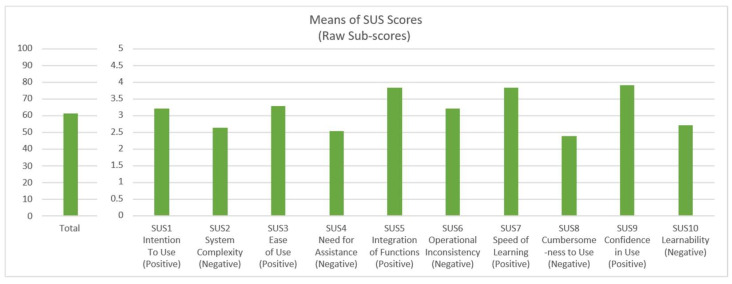
Means of SUS scores by participants for *Age of Empires II: Definitive Edition*.

The mean scores for each SUS item and the overall SUS score are presented in Figure 13. As shown in the figure, participants rated the system relatively favorably on several positive aspects. The highest-scoring items were SUS9 (“I felt very confident using the system”; M = 3.92, SD = 0.84), SUS5 (“I found the various functions in this system were well integrated”; M = 3.83, SD = 0.91), and SUS7 (“I would imagine that most people would learn to use this system very quickly”; M = 3.8, SD = 0.79). These results suggest that participants found the system’s functions coherent and felt confident in their interactions after the tutorial phase.

Conversely, the lowest-scoring items were negatively phrased statements. SUS8 (“I found the system very cumbersome to use”; M = 2.39, SD = 1.01) and SUS2 (“I found the system unnecessarily complex”; M = 2.64, SD = 0.98) received relatively lower scores, indicating that participants did not perceive the system as overly complex or cumbersome. However, SUS4 (“I think that I would need the support of a technical person to be able to use this system”; M = 2.53, SD = 1.06) and SUS10 (“I needed to learn a lot of things before I could get going with this system”; M = 2.72, SD = 1.03) scored moderately, suggesting that some participants may have felt a learning curve or desired additional support.

The overall mean SUS score was 61.46 (SD = 13.28). According to the interpretive grading scale proposed by Bangor et al. [38], a SUS score of 61.46 falls between “OK” (50.9) and “Good” (71.4), indicating an acceptable level of perceived usability. This score is also consistent with typical SUS ratings for complex software systems and aligns with previous research on electronic health record usability, where mean SUS scores often range from 45 to 65 [3]. These findings suggest that while the game system was generally usable for novice RTS players, there remains room for improvement, particularly in reducing the initial learning curve and providing more intuitive onboarding experiences.

### 3.7. Thematic Analysis of Interview Texts

The semi-structured interviews provided supplementary insights into participants’ experiences, complementing the quantitative findings. A thematic analysis identified key patterns across the reflection interviews (Experiment 4) and final interviews (all experiments), highlighting tactical understanding, operational challenges, and fatigue perceptions.

#### 3.7.1. Reflection Interview Analysis (Exp4: Germane Load Contrast)

Analysis Scope: Reflection interview sections for participants P1–P18. These interviews focused on a single experiment: controlling 10 Men-at-Arms, 10 crossbowmen, and 10 knights against multiple waves of enemy Swordsmen on a straight-line map.

- Theme 1: Unit Role and Tactical Understanding

Description: During reflection, participants generally formed a basic tactical understanding of the three units (Swordsman/Infantry, crossbowmen/archer, knight/cavalry), namely “frontline tanking” and “backline damage dealing.” This formed the basis for their improvement strategies.

Frequency: Appeared in 15 out of 18 reflection interviews.

Representative Quotes:

P2: “In terms of positioning, I think the infantry is the main combat force… and our crossbowmen are in the back.”

P24: “Swordsmen and knights are frontline melee units, tank units, while the main damage output comes from the crossbowmen in the backline. So it’s necessary to protect the crossbowmen from taking too much damage.”

P28: “It’s best to have the knights protect the archers properly, so they don’t die too much in the early stage. Later on, our attack power would be much worse.”

- Theme 2: Operational Difficulties and Intent to Improve

Description: Participants identified operational difficulties from their first round, primarily “charging in all together” which exposed the backline and caused formation chaos. Based on this, they clearly proposed specific improvement strategies for the next round, such as splitting unit control, protecting the backline, and kiting.

Frequency: Appeared in 14 out of 18 reflection interviews.

Representative Quotes:

P6: “I want to try to have the infantry charge at the front, with archers providing ranged damage from behind, and cavalry… trying to go after their archers.” (Adjusting strategy after reflection)

P8: “Strategy: enhance the protection of the backline, and at the same time, possibly refine unit selection as much as possible. Select smaller groups, allowing for more detailed operation of the three unit types.”

P36: “I selected a few crossbowmen to kite the enemy units, while letting my knights and swordsmen attack. I think this strategy is pretty good.” (Adjusting in real-time during the first round and summarizing)

- Theme 3: Observation of Enemy AI (Aggro Mechanism):

Description: Many participants keenly observed that the enemy AI tended to bypass the front line and prioritize attacking the backline crossbowmen. This observation directly influenced their defensive strategy, emphasizing the need to protect ranged units specifically.

Frequency: Appeared in 9 out of 18 reflection interviews.

Representative Quotes:

P10: “I observed that they will first attack the units at the very front. If all the frontline units are already being attacked, they will go past the frontline to hit the backline crossbowmen. Given this tactic, it really highlights the importance of protecting the crossbowmen.”

P12: “They attack the backline first, they tank the damage and just head straight for the backline. So the strategy is to pull the archers back a bit when they get chased, and let the faster cavalry chase and kill.”

P18: “I don’t know why the enemy troops choose to go around to the back. Maybe they know [the crossbowmen] have low health.”

#### 3.7.2. Final Interview Analysis (All Experiments)

Analysis Scope: Final interview sections for participants P1–P36. These interviews covered all four experiments: 1. multitasking dimension (wood cutting vs. wood cutting and defense), 2. visual complexity dimension (map exploration), 3. real-time decision pressure dimension (three-choice), and 4. germane load contrast (the final level).

- Theme 1: The Challenge of Multitasking and Attentional Allocation

Description: When comparing tasks across different dimensions, the vast majority of participants found the “multitasking dimension” (simultaneously gathering wood and building units for defense) to be the most challenging. The core difficulty lay in the need to frequently and rapidly switch attention between two completely different tasks and screen areas, leading to feelings of “neglecting one while managing the other” or being “flustered.”

Frequency: Out of 36 final interviews, 25 participants explicitly stated that the “multitasking” task was more challenging than the “three-choice” task.

Representative Quotes:

P5: “The one with gathering wood and simultaneously gathering wood and building soldiers was relatively more challenging. I think the three-choice one is simpler because it’s more singular… But for the previous task, you have to take into account both sides at the same time, and it felt like I wasn’t very proficient.”

P15: “The one with simultaneous wood cutting and unit production. Why? For me, I easily end up focusing on one thing and neglecting the other. I’d be operating on one side and forget about the other side.”

P19: “Because it felt like it required constantly shifting attention. You have to watch over here to see if the wood is cut, and also keep an eye on the enemy’s movements over there. It felt like you had to constantly switch your attention.”

- Theme 2: Strategy Formation and Time Perception Under Decision Pressure

Description: For the “real-time decision pressure” dimension (three-choice), participants generally reported that after the initial unfamiliarity, they quickly formed a stable strategy centered on the principle of safety (e.g., ensuring sufficient military force before developing the economy). With this strategy in place, the difference between the 3 s and 5 s windows became insignificant, as their decision was usually made within a couple of seconds.

Frequency: Out of 36 final interviews, 20 participants explicitly mentioned that the difference between 3 and 5 s was minimal or had no effect. Almost all participants described a similar strategy of “prioritizing military, then gathering resources.”

Representative Quotes:

P9: “For the first 3 s round, I definitely felt a bit anxious mentally… but later on, because the logic was quite simple, I just looked at what enemy unit came out and clicked the corresponding one. By the time I played the 5 s round, I basically didn’t even think about the time anymore.”

P21: “First and foremost, the most important factor is not letting the enemy in… My top priority is safety.”

P30: “For this three-choice one, I actually think the difference between 3 s and 5 s isn’t that big. Because with my strategy, I can make the decision within 3 s anyway.”

- Theme 3: Fatigue Induced by Monotony in Low-Load Tasks

Description: Although the experiment explored cognitive load, the “fatigue” or “tiredness” reported by participants was not from high-difficulty tasks, but rather from low-load, repetitive tasks. This was particularly true for the low-load version of the visual complexity dimension (map exploration) and the low-load version of the multitasking dimension (pure wood cutting). The monotony and lack of operation led to distraction and mental exhaustion.

Frequency: Out of 36 final interviews, 19 participants explicitly mentioned that the map exploration task or the pure wood-cutting task made them feel bored, drowsy, or fatigued.

Representative Quotes:

P4: “I felt a bit tired during the simple map exploration run. Oh, walking along that very winding road, I felt like I didn’t know what I could do to make it go faster… and because the map was very monotonous, it just felt very boring.”

P18: “For the exploration one, I’d get a bit bored walking in the middle… and when bored, sometimes you don’t notice in time that your unit has already reached the spot you clicked, because your attention has already wandered.”

P32: “The map exploration one easily makes you drowsy.”

- Theme 4: The Impact of Reflection on Learning Effects and Limitations in Execution

Description: In the final germane load experiment, most participants felt that the intermediate reflection session (or thinking during the break) was helpful for their second-round performance. This help was mainly reflected in clarifying strategies and adjusting tactics. However, many also pointed out a gap between the theoretical “reflected strategy” and the actual “operational execution,” i.e., “knowing what to do, but not being able to do it,” leading to limited actual improvement.

Frequency: Out of 36 final interviews, 28 participants clearly answered this question. Among them, 20 found it helpful, and 8 found it not very helpful or not helpful at all.

Representative Quotes:

P2: “I think it was helpful, but not a lot… I feel like there are some mechanics in this game that I didn’t fully understand… the later part of the fight was quite chaotic. But yeah, I could still reflect and play a bit better.”

P24: “It was a little helpful, but during the actual operation… reflection is just a theoretical thing, but ultimately, you have to rely on actual operation to decide whether the strategy is successful or effective. Yes, like that.”

P28: “I think it was very helpful, to… reflect and figure out how we should try to pass the level next time? Because if you do the same thing every time, you definitely won’t pass. You have to… look at why you failed last time and change the strategy, find out the reason for the failure, right?”

## 4. Discussion

### 4.1. Hypothesis Testing Summary

This study proposed three core hypotheses based on cognitive load theory [5,29] and the Neurovisceral Integration Model [39], predicting that increased task demands would elevate subjective workload, impair behavioral performance, and induce changes in autonomic physiological responses.

However, while the subjective and behavioral effects largely aligned with theoretical expectations, the group-level HRV differences were generally limited. Instead, physiological effects were more evident in individual-level correlations with perceived workload rather than in robust between-condition contrasts. This pattern suggests that autonomic responses during complex RTS-based tasks may reflect task-related modulation rather than uniform stress-driven activation. Accordingly, the conclusions below distinguish between condition-level effects and individual-level autonomic associations:

- H1 (Multitasking) Partially Supported: As predicted, higher levels of multitasking significantly increased subjective cognitive load (especially temporal demand and frustration) and led to poorer behavioral performance (longer completion time). These subjective–behavioral links were further supported by significant positive correlations between temporal demand, frustration, total raw-TLX, and completion time across conditions. However, physiological load, as indexed by HRV metrics, did not show significant between-group differences, despite widespread correlations with subjective load. This dissociation between subjective experience and physiological response under high multitasking demand—consistent with the broader cross-experimental pattern where sympathetic indices negatively correlated with subjective load—highlights the importance of individual differences in autonomic reactivity, while confirming that perceived workload remains tightly coupled with behavioral efficiency.
- H2 (Visual Complexity) Not Supported: The hypothesis that higher visual complexity would increase physiological load and subjective cognitive load and impair behavioral performance was not supported. Apart from a slight increase in temporal demand, there were no significant differences in subjective load dimensions, behavioral performance, or HRV metrics. Nevertheless, significant correlations between HRV indices (e.g., RMSSD, stress index) and subjective load, as well as between subjective load (e.g., mental demand, effort) and completion time, were observed across conditions. This indicates that even when the visual manipulation itself does not elicit a strong group-level effect, individual variations in perceived workload and physiological state remain associated with task efficiency—a pattern that aligns with the cross-experimental stability of global autonomic indices (LF power, SD2) as robust markers of cognitive arousal.
- H3 (Real-Time Decision Pressure) Partially Supported: Higher real-time decision pressure significantly increased subjective cognitive load (mental, physical, and temporal demands), consistent with the hypothesis. However, behavioral performance (completion time) was not impaired, and physiological load (HRV) showed only limited between-group differences. Interestingly, mental demand and physical demand correlated negatively with completion time across conditions, suggesting that participants who invested more mental effort under pressure completed the task faster—a key indicator of strategic adaptation. HRV metrics (e.g., SD2, LF power, LF/HF) also correlated significantly with subjective load, confirming that physiological measures remain sensitive to perceived time pressure even when average group differences are attenuated. Notably, the positive correlation between LF/HF and subjective load may reflect a relative shift in sympathovagal balance under time pressure, while the marginal decrease in stress index highlights the value of multi-indicator approaches in capturing distinct facets of autonomic response [2].

### 4.2. Overview of Main Findings

This study adopted a multimodal framework integrating physiological indicators (HRV), subjective workload (raw NASA-TLX), and behavioral performance to examine the effects of three cognitive load dimensions within a real-time strategy (RTS) task context, and to explore the potential impact of reflection on germane load.

Across experiments, a consistent pattern emerged: manipulations that increased task demands reliably elevated subjective workload, whereas physiological responses showed limited between-condition differences but stable associations with perceived load at the individual level.

In the multitasking dimension (Experiment 1), dual-thread tasks significantly increased subjective workload and prolonged completion time, despite the absence of significant group-level HRV differences. Nevertheless, HRV metrics correlated with subjective load, indicating individual-level autonomic modulation linked to perceived demand.

In the visual complexity dimension (Experiment 2), only temporal demand increased significantly under complex scenes, with no clear physiological or behavioral differences. However, correlations between HRV indices and subjective load persisted, suggesting subtle autonomic engagement even when overt performance changes were absent.

In the real-time decision pressure dimension (Experiment 3), shortened decision windows significantly elevated subjective workload. While group-level HRV differences were limited and the stress index showed a marginal decrease, consistent correlations between HRV metrics and subjective load were observed, reinforcing the sensitivity of physiological measures to perceived time pressure.

In the germane load contrast (Experiment 4), reflection did not produce significant differences across primary metrics, with only marginal effects on LF/HF ratio, indicating a limited but potentially specific influence on autonomic balance.

Qualitative findings supported these patterns by revealing adaptive strategies, perceived multitasking difficulty, time-pressure-driven adjustments, and constrained reflection effects, thereby providing contextual interpretation of the quantitative results.

### 4.3. Cross-Experiment Patterns: Stable Associations Across Task Types

Despite the task-specific dissociations discussed in the preceding sections, several associations between HRV metrics, subjective load, and behavioral performance remained remarkably stable across all four experiments, revealing fundamental links between autonomic activity and perceived effort in human–computer interaction.

#### 4.3.1. Sympathetic-Related Indices: A Counterintuitive Negative Association with Subjective Load

One of the most consistent—yet counterintuitive—findings was the negative correlation between sympathetic-related HRV indices and subjective workload. Across Experiments 1, 2, and 3, both the stress index and the SNS index showed significant negative correlations with multiple dimensions of the NASA-TLX (e.g., mental demand, physical demand, effort). In Experiment 4, the stress index also correlated negatively with effort, maintaining the same directional trend.

This pattern runs contrary to the traditional view that heightened sympathetic activity should accompany increased perceived demand [15]. Several explanations may account for this dissociation. First, the stress index and SNS index are composite measures that reflect not only sympathetic outflow but also baroreflex function and other regulatory mechanisms [32]. Second, because all HRV metrics in this study were expressed as relative change from baseline, the observed negative correlations may capture individual differences in autonomic reactivity: participants who showed smaller increases (or even decreases) in sympathetic activity under high load might have experienced higher subjective effort because they had to compensate through other mechanisms [13]. Third, the brief task duration (5–7 min) may have been insufficient to evoke a strong, sustained sympathetic response, whereas subjective perception of load accumulated more rapidly [14]. Taken together, these findings offer plausible mechanisms for the observed negative correlations, although they remain speculative and require further validation. Together, these findings highlight the importance of distinguishing between absolute sympathetic tone and task-evoked reactivity when interpreting HRV–workload relationships.

#### 4.3.2. Global Autonomic Indices: Robust Positive Markers of Perceived Load

In contrast to the sympathetic measures, global autonomic indices—particularly LF power and SD2—showed consistently positive correlations with subjective load across all four experiments. LF power, which reflects both sympathetic and parasympathetic influences, was significantly positively associated with multiple TLX dimensions in every task. Similarly, SD2, a non-linear measure of long-term RR interval variability exhibited uniform positive relationships with subjective load across all experiments. The LF/HF ratio, often interpreted as an index of sympathovagal balance, also correlated positively with subjective load in three out of four experiments (Exp1, 3, 4), although it failed to reach significance in the visual complexity task (Exp2).

The robustness of these associations suggests that overall autonomic arousal—captured by LF power, SD2, and LF/HF—may represent a stable correlate of perceived cognitive demand, showing consistency across task types. This aligns with the view that these metrics reflect the integrative activity of the central autonomic network, which modulates heart rate variability in response to cognitive and emotional challenges [15]. The absence of a significant LF/HF effect in Exp2 may be attributable to the purely perceptual nature of the visual complexity task, which did not require active decision making and therefore elicited less sympathovagal adjustment.

#### 4.3.3. Subjective–Behavioral Coupling: Expected Patterns in Multitasking and Visual Complexity

In Experiments 1 (multitasking) and 2 (visual complexity), higher subjective load—particularly temporal demand, frustration, and total raw-TLX—was associated with longer completion times (positive correlations). This pattern is entirely consistent with the intuitive notion that increased perceived effort impairs task efficiency, and it provides a validation baseline against which the reversed relationship observed in Experiment 3 can be contrasted. These findings also resonate with Wickens’ [4] multiple resource theory, which predicts that competition for common cognitive resources leads to performance decrements and elevated subjective workload when tasks share overlapping processing structures.

#### 4.3.4. Parasympathetic Indices: Task Dependent Rather than Stable

In contrast to the stable patterns described above, parasympathetic-related indices (RMSSD, SD1, HF power) exhibited considerable task dependency. While they showed positive correlations with subjective load in some experiments (e.g., RMSSD/SD1 in Exp1–2, HF power in Exp2–3), these associations were absent or directionally inconsistent in others (Exp4). Moreover, HF power correlated negatively with completion time only in the decision pressure task (Exp3), and PNS index correlated negatively with completion time only in the visual complexity task (Exp2). This variability suggests that vagally mediated HRV may reflect context specific processes such as attentional engagement, effortful control, or even adaptive coping, rather than serving as a uniform marker of workload across all task types.

#### 4.3.5. Summary

Taken together, the cross-experiment comparisons reveal two major stable patterns: (1) a consistent negative association between sympathetic-related indices and subjective load—a finding that challenges conventional interpretations and underscores the need to consider change score metrics and individual reactivity; and (2) robust positive correlations of global autonomic indices (LF power, SD2, LF/HF) with perceived workload, confirming their utility as general markers of autonomic arousal during cognitive tasks. These stable relationships provide a foundation for interpreting the task-specific dissociations discussed in Section 4.4.1, Section 4.4.2, Section 4.4.3 and Section 4.4.4, and they highlight the value of a multi-indicator approach in HRV research [2].

### 4.4. Task-Specific Patterns: Modulation by Task Demands

#### 4.4.1. Multitasking: Parasympathetic Specificity Amidst Subjective–Physiological Dissociation

The results of Experiment 1 revealed an interesting dissociation between subjective load and physiological responses under multitasking conditions. Although participants reported significantly higher mental demand, physical demand, temporal demand, effort, and frustration as well as longer task completion times under high-load conditions, HRV metrics showed no significant between-group differences. This finding aligns with the observation by Kosch et al. [2] that subjective and physiological measures may reflect different aspects of cognitive load and do not necessarily change synchronously.

This dissociation may have several explanations. First, the multitasking task (wood collection and unit production for defense) may primarily activate cognitive resources related to task switching and attentional allocation, the use of which may not be strongly reflected in short-term fluctuations of the autonomic nervous system [13]. Second, the duration of the experimental tasks (approximately 5–7 min) may have been insufficient to trigger significant cumulative physiological fatigue, even though participants subjectively felt pressure. Third, as RTS novices, participants may have invested more compensatory effort under high-load conditions, which maintained physiological homeostasis to some extent but led to increased subjective burden [14].

Notably, despite the absence of significant between-group differences, the significant correlations between HRV metrics and subjective load (e.g., RMSSD, SD1 positively correlated with mental demand; stress index negatively correlated with multiple TLX dimensions) indicate an intrinsic link between physiological responses and subjective perception at the individual level. The negative correlation between stress index and subjective load represents a cross-experimental pattern that recurred across all four studies; its underlying mechanisms—including the composite nature of the index, individual differences in autonomic reactivity, and the brevity of task duration—are discussed in detail in Section 4.3 [13,32,38].

Of particular interest in the multitasking context is how the parasympathetic indices (RMSSD, SD1) were significantly correlated only with mental demand, and not with other subjective dimensions such as temporal demand or frustration. This specificity suggests that in multitasking, vagal activity may be primarily coupled with cognitive demand rather than with time pressure or emotional responses. Task switching and attentional allocation may engage neural regulatory circuits linked to vagal function more directly than does the mere perception of time urgency.

Qualitative interviews further confirmed the challenge of multitasking. Twenty-five participants (69.4%) explicitly stated that the multitasking task was more challenging than the three-choice task, with the core difficulty lying in the need to frequently and rapidly switch attention between two completely different tasks and screen areas, leading to feelings of “neglecting one while managing the other” or being “flustered.” As P15 stated: “For me, I easily end up focusing on one thing and neglecting the other. I’d be operating on one side and forget about the other side.”

We acknowledge that the high-load condition in Experiment 1 involved not only task switching (wood collection and unit production) but also combat elements (defending against enemy attacks), which may have contributed to the observed increase in subjective workload. This design choice was motivated by ecological validity: in real-world RTS gameplay, multitasking rarely occurs in isolation—it is typically accompanied by unexpected threats and time pressure. If we had eliminated combat to isolate task switching, the resulting task would have resembled a laboratory-based dual-task paradigm rather than authentic RTS play, thereby limiting the generalizability of our findings. This tension between internal validity (the ability to isolate causal factors) and ecological validity (the ability to generalize to real-world settings) is a well-recognized challenge in game-based research [18,39]. Future studies could employ modified game paradigms that keep combat constant across conditions (e.g., always present but with varying task-switching demands) to better isolate the effects of multitasking while preserving ecological relevance.

This finding aligns with Wickens’ multiple resource theory, emphasizing that when tasks compete for the same cognitive resources, performance decline and increased subjective load are inevitable outcomes [4].

#### 4.4.2. Visual Complexity: Isolated Temporal Demand and the Engagement Hypothesis

The results of Experiment 2 indicated that the manipulation of visual complexity did not significantly affect behavioral performance (completion time) or most subjective load dimensions, with only temporal demand showing a significant difference. This finding partially deviates from expectations and may reflect the interaction between task characteristics and participant strategies.

The significant increase in temporal demand under high visual complexity conditions warrants further examination. Temporal demand, as assessed by the NASA-TLX, captures the perceived pace and time pressure associated with the task [9]. In this study, the addition of decorative elements in the high-load condition may have subtly distracted participants, extending their subjective sense of the time required for navigation, even though objective completion times were unaffected. This phenomenon is consistent with findings from visual search research, where increased visual clutter prolongs search durations and heightens perceptions of urgency, leading to elevated temporal workload without impacting overall efficiency [40]. Moreover, in HCI contexts, visual complexity has been shown to amplify temporal demands by competing for limited attentional resources, thereby impeding efficient information processing and creating a sense of rushed pacing [41,42]. This isolated effect on temporal demand underscores the nuanced ways in which visual design can differentially impact workload components, reinforcing the multidimensional framework of cognitive load theory [5].

Several factors may explain why visual complexity did not affect other subjective dimensions or behavioral performance. First, the map exploration task was essentially a low-cognitive-demand navigation task in which participants simply controlled a unit to follow a designated path to the endpoint. Even though the high-load version added visual decorations, the core task requirements remained unchanged, thus limiting the impact on overall performance [20]. Second, participants may have developed selective attention strategies, actively ignoring irrelevant visual information, thereby counteracting the potential interference of visual complexity. Third, the relatively short task duration (approximately 7 min) may have been insufficient for the cumulative effect of visual distraction to significantly influence physiological indicators.

We also note that the high-load condition in Experiment 2 included dynamic elements (e.g., AI units in combat) in addition to static decorative landmarks. This introduced motion-related attentional capture beyond purely static visual complexity, which may have confounded the manipulation. However, dynamic visual elements are inherent to realistic HCI environments—real-world interfaces and games contain moving elements that naturally attract attention. Excluding them entirely would have resulted in a stimulus divorced from real-world visual complexity, limiting the generalizability of our findings [43,44]. Future studies could use purely static visual manipulations (e.g., varying the density of static objects while keeping motion constant) to isolate the effect of visual complexity, or systematically vary motion to examine its independent contribution.

However, the widespread correlations between HRV metrics and subjective load (e.g., RMSSD, SD1 positively correlated with multiple TLX dimensions; stress index negatively correlated with TLX dimensions) suggest that even when visual complexity does not significantly alter behavioral performance, it may still influence users’ physiological states in subtle ways. Notably, the positive correlation between HF power and multiple subjective load dimensions indicates that parasympathetic activity may be activated in visually rich environments, possibly reflecting participants’ “engagement” or “involvement” rather than mere load increase. This observation aligns with the cross-experimental pattern of task-dependent parasympathetic associations discussed in Section 4.3: in certain contexts, vagal activation may signal positive cognitive engagement rather than relaxation or absence of load.

Qualitative interviews further support this interpretation. Several participants (e.g., P18, P34) mentioned that the complex decoration maps were “more interesting” and made them “want to explore the next scene,” while simple maps were “boring” and made them “drowsy.” This finding suggests that visual richness may alleviate monotony-induced fatigue by enhancing task engagement, rather than necessarily increasing cognitive load. This aligns with research by Zhang et al. [34] on the impact of game environments on player states, emphasizing that interface design should balance information density with user experience.

#### 4.4.3. Real-Time Decision Pressure: Strategic Adaptation Underpins the Subjective–Behavioral Dissociation

The results of Experiment 3 showed that shortening the decision window (from 5 s to 3 s) significantly increased participants’ subjective load, particularly in the mental demand, physical demand, and temporal demand dimensions, but did not significantly affect behavioral performance (completion time). This pattern reflects the interaction between time pressure and strategic adaptation. Although the 3 s window objectively reduced decision time, participants coped with the pressure by forming stable strategies (e.g., ensuring sufficient military force before developing the economy), thereby maintaining task efficiency. In qualitative interviews, 20 participants (55.6%) explicitly stated that the difference between 3 and 5 s was minimal or had no effect, as their decisions were typically made within a couple of seconds. As P9 described: “For the first 3 s round, I definitely felt a bit anxious mentally… but later on, because the logic was quite simple, I just looked at what enemy unit came out and clicked the corresponding one.”

Correlation analyses provided further quantitative support for this strategic adaptation account: across conditions, mental demand and physical demand were significantly negatively correlated with completion time (r = −0.2654, *p* = 0.0243; r = −0.2497, *p* = 0.0344, respectively). This counterintuitive finding suggests that participants who perceived higher mental and physical effort under time pressure actually completed the task faster—a pattern consistent with the notion that increased cognitive investment can enhance performance when effective strategies are in place [8]. This finding aligns with research by Ontañón et al. [22] on player strategy formation in RTS games, indicating that experience accumulation and strategy simplification are effective mechanisms for coping with time pressure.

Concurrently, the widespread correlations between HRV metrics and subjective load (e.g., SD2 and LF power positively correlated with multiple TLX dimensions) suggest that even when behavioral performance is unaffected, the physiological system still responds to time pressure at the individual level. Notably, the positive correlation between the LF/HF ratio and multiple subjective load dimensions reflects a shift in sympathovagal balance toward sympathetic dominance, consistent with the traditional understanding of time pressure [15].

Interestingly, the stress index showed a marginally significant decrease under high-load conditions (*p* = 0.0715)—a direction opposite to the expected sympathetic activation. This apparent contradiction with the LF/HF findings may reflect the specificity of different HRV indicators: while the stress index has been associated with overall autonomic strain, it may be less sensitive to the acute, time-locked nature of decision pressure compared to frequency-domain metrics. Alternatively, the marginal significance suggests the possibility of statistical fluctuation, warranting cautious interpretation. The discrepancy between the LF/HF and stress index findings highlights the importance of using a multi-indicator approach in HRV research, as different metrics may capture distinct facets of autonomic response under cognitive load [2].

Taken together, the subjective, behavioral, physiological, and qualitative findings paint a coherent picture: under real-time decision pressure, participants experienced heightened subjective load but maintained behavioral performance through strategic adaptation, with the negative subjective–behavioral correlations suggesting that mental effort, when effectively deployed, may be associated with faster task completion.

#### 4.4.4. Germane Load and Reflection Intervention: The Gap Between Theoretical Expectations and Actual Effects

Experiment 4 revealed no significant differences between the reflection and rest groups on any metric, except for a marginal decrease in the LF/HF ratio in the reflection group. This finding partially deviates from cognitive load theory’s expectation that reflection promotes schema construction [6,8], and may be explained by several factors.

First, the 5 min reflection may have been too brief. Qualitative interviews indicated that reflection was “helpful, but not a lot” (P2, P24), with a gap between theoretical strategies and actual execution—suggesting that learning effects may require longer interventions or more practice. Second, the task complexity limited reflection’s impact; controlling 30 units against multiple enemy waves demands fine motor skills unlikely to improve through short reflection alone [24,34]. Third, the rest group may have engaged in spontaneous reflection (e.g., P23 mentioned: “I actually thought a bit about why I lost the first round? I was thinking about how to win.”), narrowing between-group differences.

Despite the lack of group effects, correlation analyses revealed stable physiological–subjective associations. Consistent with cross-experiment patterns (Section 4.3), LF power and SD2 correlated positively with multiple subjective load dimensions, confirming global autonomic arousal as a robust marker of cognitive load [15].

Two distinctive findings emerged in Experiment 4. First, mean RR correlated negatively with mental demand—the only instance across experiments where mean RR significantly associated with a subjective metric—possibly reflecting individual differences in autonomic baseline post-intervention. Second, unlike previous experiments, sympathetic indices (SNS index) showed no significant correlations, and parasympathetic indices (RMSSD, SD1, and HF power) exhibited inconsistent patterns. This may stem from the use of kill count (rather than completion time) as the behavioral metric, which could be less sensitive to autonomic–behavioral coupling.

Nevertheless, the marginal LF/HF decrease in the reflection group suggests a potential positive impact on autonomic balance, promoting parasympathetic activity in line with stress recovery theories [15]. This warrants future research with longer interventions and more precise physiological monitoring.

### 4.5. Fatigue Induced by Both Overload and Underload: A U-Shaped Relationship

An unexpected but consistent theme in the qualitative interviews was that the “fatigue” or “tiredness” reported by participants came not from high-difficulty tasks, but from low-load, repetitive tasks, particularly the low-load version of the visual complexity dimension (map exploration) and the low-load version of the multitasking dimension (pure wood cutting). Nineteen participants (52.8%) explicitly mentioned that these tasks made them feel bored, drowsy, or fatigued.

This finding reveals a U-shaped relationship between cognitive load and subjective experience: both excessively high and excessively low loads can lead to negative experiences, but through different mechanisms. High load induces stress-related fatigue through resource depletion, while low load induces boredom-related fatigue through monotony.

This U-shaped interpretation is derived from participants’ qualitative reports of boredom and fatigue, rather than from direct measurement of boredom or monotony (i.e., underload). While this limits the empirical support for the interpretation, recent research has established that boredom itself is a demanding state that requires cognitive effort to sustain task engagement, and that prolonged exposure to monotonous conditions can lead to mental fatigue [45,46]. These findings lend indirect support to the notion that low-load tasks may induce fatigue through boredom-related mechanisms, complementing the well-known stress-related fatigue induced by high-load tasks. Nevertheless, future studies should directly measure boredom and monotony to empirically test this hypothesized U-shaped relationship.

As P18 described: “For the exploration one, I’d get a bit bored walking in the middle… and when bored, sometimes you don’t notice in time that your unit has already reached the spot you clicked, because your attention has already wandered.”

This finding has important implications for HCI design: in industrial applications, it is necessary not only to avoid excessively high cognitive load leading to errors and stress but also to avoid excessively low task demands leading to attention lapses and decreased vigilance [1]. Interface design should maintain user engagement through appropriate information density and interaction frequency, achieving an “optimal challenge zone.”

### 4.6. Validity of HRV as a Cognitive Load Indicator

This study comprehensively examined the sensitivity of various HRV metrics across different cognitive load dimensions. Overall, time-domain metrics (RMSSD, SD1), frequency-domain metrics (LF power, HF power), and non-linear metrics (SD2, stress index) all showed widespread correlations with subjective load, but had limited sensitivity in between-group comparisons.

This pattern aligns with the review by Kosch et al. [2], indicating that the sensitivity of HRV to cognitive load is influenced by multiple factors, including task type, duration, individual differences, and metric selection. In this study, HRV metrics may be more sensitive in reflecting intra-individual load changes (e.g., correlations with subjective load) than between-group mean differences. This aligns with research by Borisov et al. [13] on robust cognitive load detection, emphasizing the importance of multimodal fusion.

Notably, metrics such as stress index and SD2 showed stable and directionally consistent associations with subjective load across multiple experiments, suggesting these metrics may be more suitable as physiological markers of cognitive load. Meanwhile, the marginally significant change in the LF/HF ratio in Experiment 4 suggests it may be sensitive to cognitive activities such as reflection intervention. Future research should further validate the specificity and sensitivity of these indicators.

### 4.7. Perceived System Usability and Its Implications

To complement the physiological and behavioral findings, the System Usability Scale (SUS) was administered at the end of the experiment to assess participants’ overall perceived usability of the game platform. The mean overall SUS score was 61.4661.46 (SD = 13.28; see Figure 13), which, according to the interpretive grading scale proposed by Bangor et al. [38], falls between “OK” (50.9) and “Good” (71.4). This indicates an acceptable level of perceived usability for the RTS game environment employed in this study.

An analysis of individual SUS items revealed that participants rated the system most favorably on items related to confidence (SUS9: M = 3.92), functional integration (SUS5: M = 3.83), and ease of learning for others (SUS7: M = 3.83). These findings suggest that after the tutorial phase, participants developed a reasonable mental model of the system’s functionality and felt adequately confident to perform the required tasks. This is particularly important given that all participants were RTS novices; the acceptable usability ratings help validate the experimental platform as one that did not introduce excessive extraneous cognitive load solely due to poor interface design [27].

Conversely, the relatively moderate scores on items related to the need for technical support (SUS4: M = 2.53) and the amount of learning required (SUS10: M = 2.72) suggest that some participants experienced a learning curve, particularly during the initial stages of interaction. This observation aligns with qualitative reports from several participants (e.g., P23, P29) who mentioned that they felt “overwhelmed” or “confused” during the first few minutes of gameplay. These findings highlight an important consideration for the design of training protocols in complex HCI environments: while the system may be ultimately usable, the initial onboarding experience plays a crucial role in shaping users’ perceived workload and confidence.

The SUS score of 61.46 is also consistent with typical ratings for complex software systems, including those reported in studies of electronic health record usability [3], where mean scores often range from 45 to 65. This convergence suggests that the RTS platform used in this study provides a valid and ecologically relevant simulation environment for investigating cognitive load in complex HCI contexts. The acceptable usability ratings help mitigate concerns that the observed cognitive load effects might be artifacts of poor system design rather than reflections of the intrinsic cognitive demands of multitasking, visual complexity, and decision pressure.

### 4.8. Integrative Significance of Qualitative Findings

The qualitative findings extended the quantitative results by providing contextual explanations for the stable correlations observed between HRV metrics and perceived workload across experiments. Rather than merely corroborating statistical outcomes, the interviews clarified how participants experienced and regulated cognitive demands under different task conditions.

First, unit role understanding and observation of enemy AI illustrated adaptive strategy formation. Participants reported actively adjusting positioning and control strategies in response to task demands (e.g., anticipating enemy targeting patterns). Such adaptive engagement may help explain why physiological indices were associated with subjective workload even when group-level differences were limited, as autonomic modulation may reflect individual differences in strategic involvement rather than uniform stress responses.

Second, operational difficulties and explicit intent to improve highlighted metacognitive monitoring processes during reflection. Participants identified execution errors and proposed corrective strategies, reflecting ongoing schema refinement. This aligns with cognitive load theory in emphasizing structured knowledge construction, while also illustrating how subjective effort may coexist with performance stability through adaptive regulation.

Third, fatigue reported in low-load conditions revealed that reduced task complexity did not necessarily correspond to improved experiential states. Reports of monotony-induced tiredness suggest that perceived workload is multidimensional, encompassing both overload and under-stimulation. This may further account for the nuanced associations between physiological measures and subjective ratings observed across conditions.

### 4.9. Limitations and Future Directions

This study has several limitations. First, the sample consisted of young university students (20–28 years old), primarily RTS novices, which may limit the generalizability of the findings. Future research should include participants of different ages, professional backgrounds, and experience levels to verify the universality of the findings.

Second, HRV measurement is influenced by multiple factors (e.g., respiration, movement, emotion). Although standardized procedures and strict data preprocessing were employed, confounding variables may still exist. Future research could combine multimodal physiological measurements (e.g., eye tracking, electrodermal activity, electroencephalography) to enhance measurement reliability.

Third, the duration of experimental tasks was relatively short (5–7 min), which may have been insufficient to trigger significant cumulative physiological fatigue. Future research could design longer-duration tasks to examine the cumulative effects of cognitive load and fatigue development trajectories.

Fourth, the reflection intervention lasted only 5 min, which may have been insufficient to produce significant learning effects. Future research could extend intervention time and increase the depth of intervention (e.g., providing specific strategic guidance) to more effectively promote schema construction.

Fifth, the qualitative analysis employed AI-assisted methods with manual review. A formal inter-coder reliability check (Section 2.7) showed substantial agreement for reflection interviews (κ = 0.58) and final interviews (κ = 0.92). Given the exploratory nature, findings should be interpreted as hypothesis-generating. Future research could use larger coding samples and multiple independent coders with specialized training.

Sixth, the transferability of findings from game-based research to real-world contexts is a recognized challenge [19]. Factors such as time constraints, lack of practice, and established cognitive patterns may hinder effective transfer. Accordingly, the present findings—particularly the absence of significant group-level HRV differences—should be interpreted with caution when generalizing to industrial applications.

Seventh, this study relied solely on HRV as the physiological measure of cognitive load. While HRV provides valuable insight, it may not capture the full spectrum of cognitive dynamics as effectively as multimodal approaches (e.g., EEG, EDA) [47,48]. The limited discriminative power of HRV at the group level—despite its significant individual-level correlations—may partly reflect this monomodal limitation. Future research should incorporate multimodal physiological sensing.

Eighth, the sample size (*n* = 36) was determined a priori using G*Power (dz = 0.5, α = 0.05, power = 0.80), which indicated a required sample size of 34 (Section 2.2). While the analysis of 11 HRV indices might raise concerns about multivariate power, these indices are highly co-linear (e.g., RMSSD and SD1 are mathematically equivalent). Our analytical strategy focused on cross-experiment consistency patterns (Table 2) rather than formal multivariate modeling. This approach is consistent with recent HRV literature, which emphasizes that in exploratory studies, sample sizes of 30–50 are common, and that effect sizes and replicable patterns should be prioritized over isolated *p*-values [49,50]. Future research with larger samples could employ multivariate methods to further validate these patterns.

### 4.10. Theoretical Contributions, Practical Implications, and Cross-Domain Applications

The theoretical contributions of this study are primarily manifested in three aspects. First, by systematically manipulating three cognitive load dimensions—multitasking, visual complexity, and real-time decision pressure—within a controlled RTS game environment, it validated the applicability of cognitive load theory [5,29] in complex, dynamic HCI contexts, extending the boundaries of the theory’s application beyond traditional instructional design. Second, it revealed the complex relationship between subjective load and physiological responses across different load dimensions. The dissociation observed in multitasking (subjective load increased without significant group-level HRV changes) and the isolated increase in temporal demand under visual complexity underscore the importance of multimodal assessment [2,13] for capturing the full spectrum of cognitive load. The widespread correlations between HRV metrics (e.g., RMSSD, stress index, LF/HF) and subjective NASA-TLX dimensions further validate HRV as a sensitive physiological marker of perceived workload, particularly for intra-individual variations. Third, this study identified the phenomenon of monotony-induced fatigue in low-load tasks, proposing a U-shaped relationship between cognitive load and subjective experience. This finding extends CLT by highlighting that both excessively high and excessively low loads can lead to negative outcomes—stress-related fatigue versus boredom-related fatigue—emphasizing the need to consider load directionality in task design.

On a practical level, this study offers multiple insights for industrial HCI design. First, multitasking interface design should minimize the need for frequent attention switching. This can be achieved through task integration, information grouping, and visual cues to reduce switching costs, thereby mitigating the subjective burden observed in Experiment 1 [4]. Second, visual information design should balance information density with user experience. The finding that visual complexity significantly increased temporal demand without affecting objective performance (Experiment 2) demonstrates that designers must consider not only efficiency but also users’ subjective time pressure. Interfaces should avoid both excessive density (leading to cognitive overload) and insufficient density (leading to attention lapses and boredom). Third, time pressure management should account for users’ strategic adaptation capabilities. Experiment 3 showed that participants maintained performance under high decision pressure by forming stable strategies (e.g., prioritizing military production before economic development). Providing appropriate decision support—such as predictive cues or default options—can alleviate subjective burden while preserving these adaptive strategies. Fourth, training and reflection design should combine practice with structured reflection. The “knowing–doing gap” identified in Experiment 4 suggests that brief reflection interventions are insufficient for complex skill acquisition; instead, simulated practice with immediate feedback may be necessary to promote skill internalization. Fifth, fatigue monitoring and intervention should consider the directionality of load, distinguishing between stress-related fatigue (from overload) and boredom-related fatigue (from underload), and designing targeted interventions accordingly.

Crucially, the cognitive load dimensions manipulated in this study—multitasking, visual complexity, and real-time decision pressure—are not unique to RTS games. They represent common challenges in many high-stakes, complex human–computer interaction domains, such as air traffic control, emergency medicine, and industrial process control [1,4]. Therefore, the findings obtained through our multimodal approach offer significant insights for understanding and optimizing complex system design in the real world.

Table 3 systematically illustrates how the findings from this study can be transferred and applied to five typical high-workload industrial scenarios. The table clarifies the commonality between each scenario and the core challenges of RTS games. It further specifies how the subjective raw NASA-TLX workload dimensions and the objective HRV physiological metrics employed in this study can be translated into assessment tools and design optimization directions for each specific context. This framework aims to provide HCI researchers, system designers, and industry practitioners with an evidence-based reference for cognitive engineering, facilitating the creation of safer, more efficient, and more human-centered interfaces.

## 5. Conclusions

This study systematically examined three cognitive load dimensions in real-time strategy (RTS) games using a multimodal framework integrating wearable HRV monitoring, subjective assessment, behavioral metrics, and qualitative analysis. Multitasking and decision pressure significantly increased subjective workload, while visual complexity primarily elevated temporal demand without substantially impairing objective performance. Decision pressure was associated with preserved performance accompanied by negative subjective–behavioral correlations, suggesting adaptive strategic regulation. Reflection intervention yielded limited autonomic modulation.

Beyond condition-based comparisons, correlational analyses across experimental contexts revealed consistent physiological–subjective coupling patterns. Although HRV indices did not reliably differentiate task conditions at the group level, several parameters demonstrated stable associations with perceived workload across individuals. Notably, the relationships were non-uniform: certain sympathetic-related indices exhibited inverse correlations with subjective ratings, whereas global variability metrics (e.g., LF power, SD2, LF/HF) showed positive associations with perceived cognitive activation. These findings indicate that physiological responses reflect individual workload appraisal rather than simple condition-driven stress intensity.

Overall, the results highlight the multidimensional and context-sensitive nature of cognitive load in complex interactive environments. The study underscores the value of multimodal physiological assessment for capturing intra-individual workload variation and provides transferable insights for cognitive engineering and interface optimization in high-demand human–computer interaction domains.

## Figures and Tables

**Table 1:** Indices for evaluating autonomic nervous system balance.

Indicator	Unit	Domain/Type	Description/Physiological Meaning
Mean RR	ms	Time	Average interval between consecutive R-R peaks; reflects overall heart rate stability, with longer intervals indicating parasympathetic dominance.
RMSSD	ms	Time	Root mean square of successive R-R interval differences; measures short-term variability, strongly linked to parasympathetic (vagal) activity.
SD1	ms	Non-linear	Standard deviation perpendicular to the Poincaré plot line-of-identity; captures short-term HRV, correlated with RMSSD and parasympathetic tone.
PNS index	-	Composite	Parasympathetic nervous system index (derived from Mean RR, RMSSD, SD1); indicates relaxation/regeneration level, higher values suggest better recovery.
Mean HR	bpm	Time	Average heart rate; higher values often signal sympathetic activation and stress.
Stress index	-	Composite	Baevsky’s stress index (based on R-R interval histogram); quantifies sympathetic dominance and overall stress, higher values indicate tension.
SD2	ms	Non-linear	Standard deviation along the Poincaré plot line-of-identity; reflects long-term HRV, including both sympathetic and parasympathetic influences.
SNS index	-	Composite	Sympathetic nervous system index (derived from SD2); measures activation/stress response, higher values suggest sympathetic dominance.
LF power	ms^2^ (FFT)	Frequency	Low-frequency power (0.04–0.15 Hz); associated with baroreflex and mixed sympathetic/parasympathetic activity.
HF power	ms^2^ (FFT)	Frequency	High-frequency power (0.15–0.4 Hz); primarily reflects parasympathetic activity and respiratory sinus arrhythmia.
LF/HF	- (FFT)	Frequency	Ratio of LF to HF power; traditionally indicates sympathovagal balance (higher ratio suggests shift in autonomic balance), though interpretation is debated.

**Table 2:** Summary of significant correlations across experiments.

HRV/Behavioral Measure		Relationship with Subjective Load	Relationship with Behavioral Performance	Cross-Experiment Pattern
Sympathetic-Related Indices	Stress index	Negative (Exp1, 2, 3, 4 †)	NS	Consistent negative association with subjective load across all tasks
	SNS index	Negative (Exp1, 2, 3)	NS	Negative in three experiments; absent in Exp4
Parasympathetic-Related Indices	RMSSD/SD1	Positive (Exp1, 2)	NS	Positive in multitasking and visual complexity; absent in decision pressure and germane load
	HF power	Positive (Exp2, 3)	Negative (Exp3)	Positive in visual complexity and decision pressure; negative with behavior only in Exp3
	PNS index	NS	Negative (Exp2)	Only in Exp2: higher PNS index linked to faster completion
Global Autonomic Indices	LF power	Positive (Exp1, 2, 3, 4)	NS	Consistently positive across all four experiments
	SD2	Positive (Exp1, 2, 3, 4)	NS	Consistently positive across all four experiments
	LF/HF	Positive (Exp1, 3, 4)	NS	Positive in three experiments; absent in Exp2
Mean RR/Mean HR	Mean RR	Negative (Exp4)	NS	Negative only in Exp4; no significant associations in other experiments
	Mean HR	Positive (Exp4)	NS	Mirror of mean RR
Subjective–Behavioral	Mental demand	—	Positive (Exp1, 2)/Negative (Exp3)	Task-dependent: Positive in multitasking & visual complexity, negative under time pressure
	Physical demand	—	Positive (Exp1, 2)/Negative (Exp3)	Same pattern as mental demand
	Temporal demand	—	Positive (Exp1)	Positive only in Exp1
	Frustration	—	Positive (Exp1, 2)	Positive in Exp1 and Exp2
	Total raw-TLX	—	Positive (Exp1)/NS (Exp2, 3, 4)	Positive only in Exp1
Physiological–Behavioral	PNS index	—	Negative (Exp2)	Faster completion linked to higher PNS in visual complexity
	HF power	—	Negative (Exp3)	Faster completion linked to higher HF under decision pressure

Note: NS = not significant; † stress index showed negative trends in Exp4 but only reached significance for effort. “Positive” means higher HRV (or subjective rating) associated with higher subjective load or longer completion time; “negative” means the opposite. For a sensitivity analysis applying a Bonferroni-type correction to key correlation patterns, see Table A5.

**Table 3:** Applicability and transferability of RTS-based HCI cognitive load research across different industrial scenarios.

Industrial Scenario	Transferability of RTS-HCI Research (Based on Four Experiments)	Subjective Load Transfer (Raw NASA-TLX)	Objective Load Transfer (HRV + Behavior)	Example Application & Potential Value
Air Traffic Control (ATC)	Multitasking (Exp1): ATC requires simultaneous radar monitoring (visual) and pilot communication (auditory) while handling emergencies, similarly to RTS “gather + combat.” Can guide the design of integrated multi-screen information displays.	Temporal Demand, Frustration: Use TLX to assess controller stress during peak hours, design intuitive alert prioritization interfaces to reduce frustration.	HRV (RMSSD, LF/HF), Behavioral: Real-time HRV monitors sympathetic activation, predicts fatigue thresholds, enabling early warning systems.	ATC Simulator Training: Employ RTS-like multitasking interfaces with HRV biofeedback to reduce burnout risk among controllers.
Emergency/Operating Room	Real-Time Decision Pressure (Exp3): Fast triage and intraoperative decisions under time pressure mirror RTS’s time-limited production choices.	Mental Demand, Effort: Quantitatively assess different EMR interface designs, simplifying information presentation to reduce “information overload.”	HRV (HF Power, Stress Index): Objectively track stress peaks during critical surgical steps, integrate into wearables for real-time alerts.	Smart OR/EMR Design: Leverage RTS decision-support logic to optimize information flow, reduce medical errors, and improve clinician well-being.
Military Command & Control (C2)	Visual Complexity (Exp2): Modern C2 systems have high visual clutter from multi-source data; requires rapid threat detection in complex scenes.	Effort, Frustration: Subjective load assessment guides the design of low-clutter command interfaces, reducing the commander’s information filtering burden.	HRV (SDNN, LF/HF), Behavioral: Monitor operator physiological state under high load, predict cognitive breakdown points, optimize human–machine teaming.	Next-Gen C2 Systems: Use RTS cognitive models to train personnel, improving decision speed and accuracy in complex battlefield environments.
High-Frequency Trading	Decision Pressure + Multitasking (Exp1, 3): Traders monitor massive multi-screen data and make split-second decisions during market volatility.	Temporal Demand, Effort: Use TLX to evaluate trading platform usability, design AI tools to filter noise, allowing focus on core decisions.	HRV (RMSSD, SD2): Objectively measure decision load during market stress, develop biofeedback apps to help traders maintain composure.	Trading Platform Ergonomics: Apply RTS-HCI findings to optimize trading software interfaces with HRV monitoring to prevent trader burnout.
Industry 4.0/Process Control	Multitasking + Visual Complexity (Exp1, 2): DCS operators monitor hundreds of sensors and alarms, analogous to managing resources and unexpected threats in RTS.	Mental Demand, Frustration: Subjective load scales guide the simplification of SCADA dashboards, reducing operator “vigilance fatigue.”	HRV (Stress Index), Behavioral: Track operator physiology during prolonged low-load (monotony) and sudden high-load events, integrate into safety alerts.	Nuclear/Chemical Plant Training: Use RTS simulators to train operators for abnormal situations, combined with HRV to assess coping capacity, enhancing industrial safety.

## Data Availability

The data presented in this study are available on request from the corresponding author due to privacy and ethical restrictions.

## Supplementary Materials

The following supporting information can be downloaded at https://www.mdpi.com/article/10.3390/s26072181/s1, Figure S1: Scenario for Experiment2-l (HD); Figure S2: Scenario for Experiment2-h (HD); Figure S3: Scenario for Experiment4 (HD).

## Abbreviations

The following abbreviations are used in this manuscript:

HRV	Heart Rate Variability
RMSSD	Root Mean Square of Successive Differences
LF/HF	Low Frequency to High Frequency Ratio
SD2	Poincaré Plot Long-Term Variability
NASA-TLX	NASA Task Load Index
RTS	Real-Time Strategy
HCI	Human–Computer Interaction

## Appendix A

**Table A1:** Statistical analysis results for Experiment 1: high- vs. low-load conditions in multitasking dimension (*n* = 36).

Indicator	L Group (Mean ± SD or Median)	H Group (Mean ± SD or Median)	Statistic (t or W)	*p*-Value	Significant	Marginally Significant	Effect Size
Mean RR (%) (ms)	2.41 ± 5.88	3.03 ± 5.68	1.04	0.3034	No	No	0.17
RMSSD (%) (ms)	5.88	7.4	292	0.5291	No	No	−0.11
SD1 (%) (ms)	5.88	7.4	292	0.5291	No	No	−0.11
PNS Index (%)	−0.25	−10.53	232	0.1152	No	No	−0.26
Mean HR (%) (bpm)	−2.04 ± 5.72	−2.65 ± 5.42	−1.03	0.3081	No	No	−0.17
Stress Index (%)	−5.32	2.35	286	0.4697	No	No	−0.12
SD2 (%) (ms)	1.32	−3.64	256	0.2325	No	No	−0.2
SNS Index (%)	−8.83	−11.82	308	0.7037	No	No	−0.07
LF Power (%) (ms^2^, FFT)	2.81	22.03	294	0.5497	No	No	−0.1
HF Power (%) (ms^2^, FFT)	20.52	2.77	290	0.5089	No	No	−0.11
LF/HF (%) (FFT)	−3.54	−5.38	300	0.6137	No	No	−0.09
Mental Demand	3.5	6	23.5	0	Yes	N/A	−0.81
Physical Demand	2	4	52	0.0005	Yes	N/A	−0.74
Temporal Demand	2	6	31	0.0001	Yes	N/A	−0.79
Performance	6	8	134.5	0.0089	Yes	N/A	−0.52
Effort	2	5.5	54.5	0.0001	Yes	N/A	−0.73
Frustration	1	4	24.5	0.0003	Yes	N/A	−0.81
Total Raw-TLX	22.50 ± 14.65	36.47 ± 20.19	5.11	0	Yes	N/A	0.85
Behavioral Indicator	317.17 ± 37.26	354.92 ± 50.70	4.77	0	Yes	N/A	0.8

Note: Normality *p*-value based on Shapiro–Wilk test (*p* > 0.05 indicates normality); effect size symbols: Cohen’s d for paired *t*-test and r for Wilcoxon signed-rank test. For *t*-test: mean (SD); for Wilcoxon: median (SD). Marginally significant indicates *p* > 0.05 but *p* < 0.1.

**Table A2:** Statistical analysis results for Experiment 2: high- vs. low-load conditions in visual complexity dimension (*n* = 36).

Indicator	L Group (Mean ± SD or Median)	H Group (Mean ± SD or Median)	Statistic (t or W)	*p*-Value	Significant	Marginally Significant	Effect Size
Mean RR (%) (ms)	2.78 ± 5.83	3.43 ± 5.66	t(35) = 1.36	0.1824	No	No	0.23
RMSSD (%) (ms)	−1.79	0.54	W = 307, Z = −0.41	0.6829	No	No	−0.05
SD1 (%) (ms)	−1.81	0.5	W = 307, Z = −0.41	0.6829	No	No	−0.05
PNS Index (%)	−9.46	−7.58	W = 278, Z = −0.61	0.5445	No	No	−0.07
Mean HR (%) (bpm)	−2.40 ± 5.63	−3.04 ± 5.33	t(35) = −1.37	0.1789	No	No	−0.23
Stress Index (%)	0.68	3.23	W = 294, Z = −0.61	0.5401	No	No	−0.07
SD2 (%) (ms)	−12.67	−4.39	W = 330, Z = −0.05	0.9624	No	No	−0.01
SNS Index (%)	−13.63	−15.34	W = 267, Z = −1.04	0.2998	No	No	−0.12
LF Power (%) (ms^2^, FFT)	−16.16	−7.28	W = 251, Z = −1.29	0.1977	No	No	−0.15
HF Power (%) (ms^2^, FFT)	9.55	3.91	W = 314, Z = −0.30	0.7653	No	No	−0.04
LF/HF (%) (FFT)	−23.73	−18.92	W = 257, Z = −1.19	0.2325	No	No	−0.14
Mental Demand	1.5	2	W = 61, Z = −0.37	0.7131	No	No	−0.04
Physical Demand	2	2	W = 72, Z = −0.59	0.552	No	No	−0.07
Temporal Demand	1	2	W = 20, Z = −2.02	0.043	Yes	N/A	−0.24
Performance	4	4	W = 64, Z = −0.57	0.5679	No	No	−0.07
Effort	2	2	W = 148, Z = −0.40	0.6896	No	No	−0.05
Frustration	0	0	W = 32, Z = −1.35	0.1779	No	No	−0.16
Total Raw-TLX	13	16	W = 228, Z = −0.40	0.6875	No	No	−0.05
Behavioral Indicator	420.5	419	W = 251, Z = −0.53	0.5974	No	No	−0.06

Note: Normality *p*-value based on Shapiro–Wilk test (*p* > 0.05 indicates normality); effect size symbols: Cohen’s d for paired *t*-test and r for Wilcoxon signed-rank test. For *t*-test: mean (SD); for Wilcoxon: median (SD). Marginally significant indicates *p* > 0.05 but *p* < 0.1.

**Table A3:** Statistical analysis results for Experiment 3: high- vs. low-load conditions in real-time decision pressure dimension (*n* = 36).

Indicator	L Group (Mean ± SD or Median)	H Group (Mean ± SD or Median)	Statistic (t or W)	*p*-Value	Significant	Marginally Significant	Effect Size
Mean RR (%) (ms)	2.89 ± 5.99	2.95 ± 5.59	t = 0.11	0.9121	No	No	0.02
RMSSD (%) (ms)	5.33	9.98	W = 297.00	0.5813	No	No	0.55
SD1 (%) (ms)	5.33	9.95	W = 296.00	0.5707	No	No	0.56
PNS Index (%)	−17.97	−16.41	W = 301.00	0.6247	No	No	0.55
Mean HR (%) (bpm)	−2.49 ± 5.78	−2.59 ± 5.22	t = −0.21	0.8384	No	No	−0.03
Stress Index (%)	−3.69 ± 18.29	−8.75 ± 15.81	t = −1.86	0.0715	No	Yes	−0.31
SD2 (%) (ms)	−3.3	−0.29	W = 290.00	0.5089	No	No	0.56
SNS Index (%)	−20.58	−27.09	W = 280.00	0.414	No	No	0.58
LF Power (%) (ms^2^, FFT)	11.97	6.48	W = 278.00	0.3964	No	No	0.58
HF Power (%) (ms^2^, FFT)	23.55	34.88	W = 249.00	0.192	No	No	0.63
LF/HF (%) (FFT)	−2.72	−17.38	W = 245.00	0.1713	No	No	0.63
Mental Demand	4	5	W = 43.00	0.0019	Yes	N/A	0.94
Physical Demand	3	4	W = 61.00	0.0281	Yes	N/A	0.91
Temporal Demand	2.5	3	W = 88.00	0.0254	Yes	N/A	0.87
Performance	6.11 ± 4.00	6.92 ± 4.44	t = 1.45	0.1553	No	No	0.24
Effort	4	5	W = 162.00	0.5105	No	No	0.76
Frustration	1	1.5	W = 54.00	0.0538	No	Yes	0.92
Total Raw-TLX	20	28	W = 142.50	0.008	Yes	N/A	0.79
Behavioral Indicator	370.5	369.5	W = 272.00	0.6625	No	No	0.59

Note: Normality *p*-value based on Shapiro–Wilk test (*p* > 0.05 indicates normality); effect size symbols: Cohen’s d for paired *t*-test and r for Wilcoxon signed-rank test. For *t*-test: mean (SD); for Wilcoxon: median (SD). Marginally significant indicates *p* > 0.05 but *p* < 0.1.

**Table A4:** Statistical analysis results for Experiment 4: rest vs. reflection groups in the germane load contrast (*n* = 36).

Variable	Rest Group (Mean ± SD or Median)	Reflection Group (Mean ± SD or Median)	Statistic t (or U and Z)	*p*-Value	Significant?	Marginally Significant?	Effect Size
Mean RR (%) (ms)	−1.06	1.03	U = 198.00, Z = 1.14	0.261	No	No	0.19
RMSSD (%) (ms)	−0.68	3.42	U = 174.00, Z = 0.38	0.716	No	No	0.06
SD1 (%) (ms)	−0.65	3.38	U = 174.00, Z = 0.38	0.716	No	No	0.06
PNS Index (%)	−7.03	−2.48	U = 180.00, Z = 0.57	0.58	No	No	0.09
Mean HR (%) (bpm)	1.07	−1.02	U = 126.00, Z = −1.14	0.261	No	No	0.19
Stress Index (%)	0.64 ± 11.80	0.14 ± 20.85	t = −0.09	0.931	No	No	−0.03
SD2 (%) (ms)	2.03 ± 14.97	0.19 ± 20.46	t = −0.31	0.76	No	No	−0.1
SNS Index (%)	0.08	2.75	U = 177.00, Z = 0.47	0.646	No	No	0.08
LF Power (%) (ms^2^, FFT)	15.58	11.41	U = 142.00, Z = −0.63	0.537	No	No	0.11
HF Power (%) (ms^2^, FFT)	7.86 ± 50.66	39.94 ± 66.50	t = 1.63	0.113	No	No	0.54
LF/HF (%) (FFT)	7.37	−21.77	U = 106.00, Z = −1.77	0.079	No	Yes	0.3
Mental Demand	0	1	U = 191.00, Z = 0.92	0.362	No	No	0.15
Physical Demand	0	1	U = 193.50, Z = 1.00	0.293	No	No	0.17
Temporal Demand	0	1	U = 182.00, Z = 0.63	0.531	No	No	0.11
Performance	−1.61 ± 4.05	−1.78 ± 2.26	t = −0.15	0.88	No	No	−0.05
Effort	0	2	U = 185.50, Z = 0.74	0.457	No	No	0.12
Frustration	1	0	U = 126.00, Z = −1.14	0.257	No	No	0.19
Total Raw-TLX	1.28 ± 7.47	3.44 ± 11.59	t = 0.67	0.509	No	No	0.22
Behavioral Indicator	13.06 ± 70.12	20.78 ± 52.56	t = 0.37	0.711	No	No	0.12

Note: Rest group/reflection group normality *p* based on Shapiro–Wilk test (*p* > 0.05 indicates normality); effect size symbols: Cohen’s d for independent samples *t*-test and r for Mann–Whitney U test. For *t*-test: mean (SD); for Mann–Whitney: median (SD). Marginally significant? indicates *p* > 0.05 but *p* < 0.1.

**Table A5:** Sensitivity analysis: Bonferroni-type correction for key correlation patterns.

Key Observation	Correlation Pair	Exp1	Exp2	Exp3	Exp4	Proportion Significant
1. Sympathetic-related indices	Stress index vs. total TLX (or effort ^+^)	r = −0.3736	r = −0.3398	r = −0.3952	r = −0.2979 ^+^	2/4 (50%)
		*p* = 0.0012	*p* = 0.0035	*p* = 0.0006	*p* = 0.0110	
		p_corr_ = 0.0312	p_corr_ = 0.0910	p_corr_ = 0.0156	p_corr_ = 0.2860	
		✓	✗	✓	✗	
2. Global autonomic indices	LF power vs. total TLX	r = 0.3879	r = 0.2785	r = 0.5304	r = 0.4151	3/4 (75%)
		*p* = 0.0008	*p* = 0.0178	*p* < 0.0001	*p* = 0.0003	
		p_corr_ = 0.0208	p_corr_ = 0.4628	p_corr_ < 0.0026	p_corr_ = 0.0078	
		✓	✗	✓	✓	
	SD2 vs. total TLX	r = 0.3507	r = 0.2870	r = 0.4249	r = 0.2491	1/4 (25%)
		*p* = 0.0025	*p* = 0.0145	*p* = 0.0002	*p* = 0.0349	
		p_corr_ = 0.0650	p_corr_ = 0.3770	p_corr_ = 0.0052	p_corr_ = 0.9074	
		✗	✗	✓	✗	
	LF/HF vs. total TLX	—	—	r = 0.3091	r = 0.3484	0/4 (0%)
				*p* = 0.0082	*p* = 0.0027	
				p_corr_ = 0.2132	p_corr_ = 0.0702	
				✗	✗	
3. Parasympathetic indices	RMSSD/SD1 vs. mental demand	r = 0.3037	r = 0.3128	—	—	0/4 (0%)
		*p* = 0.0095	*p* = 0.0075			
		p_corr_ = 0.2470	p_corr_ = 0.1950			
		✗	✗			
	HF power vs. total TLX	—	r = 0.4569	r = 0.2895	—	1/4 (25%)
			*p* = 0.0001	*p* = 0.0136		
			p_corr_ = 0.0026	p_corr_ = 0.3536		
			✓	✗		
4. Subjective–behavioral relationships	Total TLX vs. completion time	r = 0.2687	—	r = −0.1042	—	0/4 (0%)
		*p* = 0.0225		*p* = 0.3837		
		p_corr_ = 0.5850		p_corr_ = 1.000		
		✗		✗		
	Mental demand vs. completion time	—	r = 0.3097	r = −0.2654	—	0/4 (0%)
			*p* = 0.0081	*p* = 0.0243		
			p_corr_ = 0.2106	p_corr_ = 0.6318		
			✗	✗		
	Physical demand vs. completion time	—	r = 0.2800	r = −0.2497	—	0/4 (0%)
			*p* = 0.0172	*p* = 0.0344		
			p_corr_ = 0.4472	p_corr_ = 0.8944		
			✗	✗		
5. Physiological–behavioral links	PNS index vs. completion time	—	r = −0.2480	—	—	0/4 (0%)
			*p* = 0.0357			
			p_corr_ = 0.9282			
			✗			
	HF power vs. completion time	—	—	r = −0.2366	—	0/4 (0%)
				*p* = 0.0454		
				p_corr_ = 1.000		
				✗		

^+^ In Experiment 4, the stress index was significantly correlated with effort (a TLX subscale) rather than total TLX; the negative direction is consistent with the other experiments. Footnote: Bonferroni correction was applied to the 26 key correlation pairs listed in this table (those explicitly mentioned in the Key Observations). Corrected significance threshold: α = 0.05/26 ≈ 0.0019. Corrected *p*-values are calculated as p_corr_ = *p* × 26, capped at 1.0. ✓ indicates *p* < 0.0019; ✗ indicates *p* ≥ 0.0019. Dash (—) indicates that the correlation was not significant in the original analysis for that experiment (and therefore not included in the correction) or was not applicable due to the experimental design. Proportion significant is defined as the number of experiments in which the correlation remained significant after correction, divided by the number of experiments in which it was tested (denominator shown). This sensitivity analysis is intended to assess the robustness of the key findings against Type I error and does not constitute a formal correction for the full set of exploratory correlations.
