# Supplementary figures and images for: HRV-Based Multimodal Physiological Signal Monitoring Using Wearable Biosensors in Human–Computer Interaction: Cognitive Load in Real-Time Strategy Games
Source: Sensors (Basel). 2026 Apr 1;26(7):2181. doi: 10.3390/s26072181 (PMC13074747; doi:10.3390/s26072181)

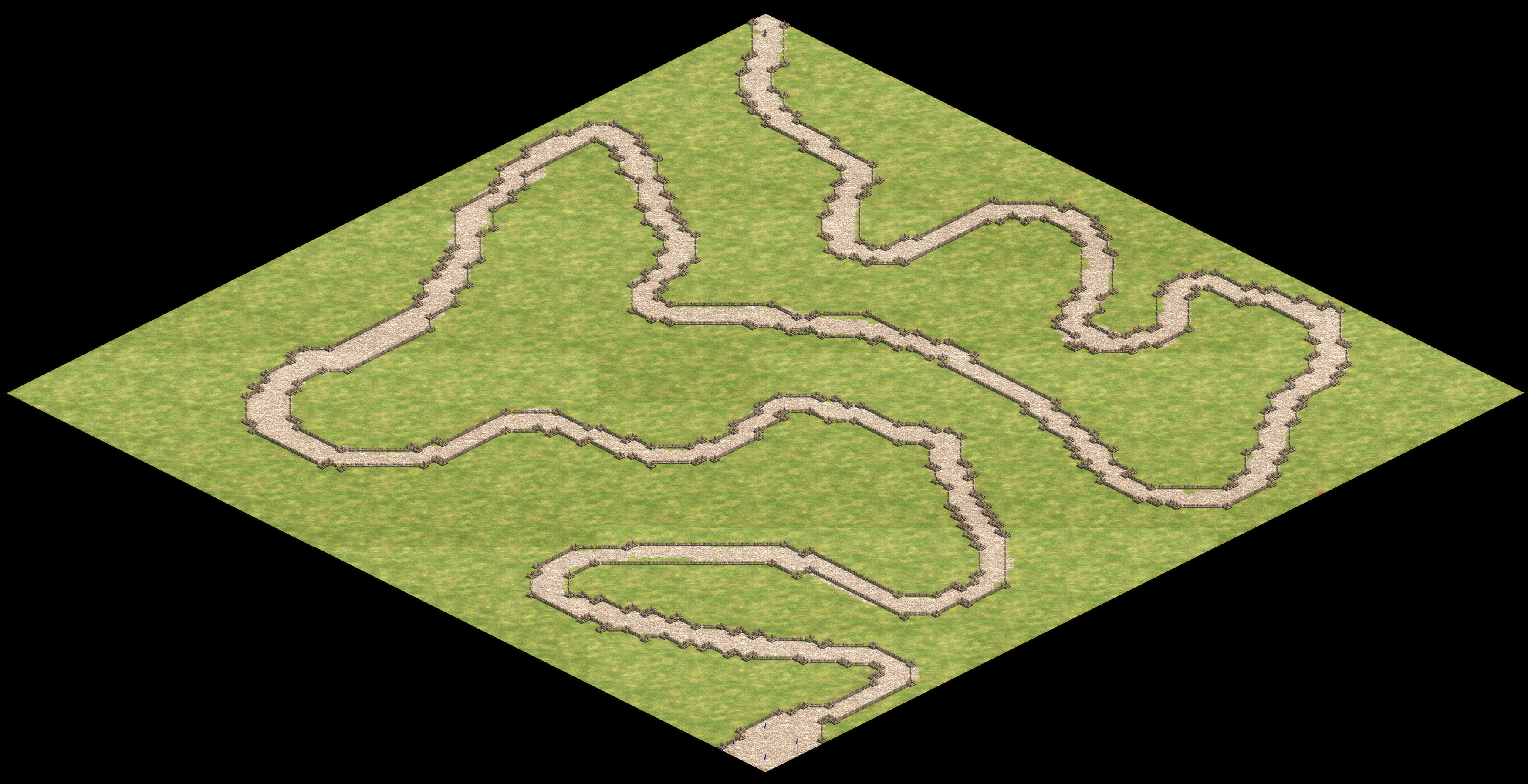

Supplement: Supplementary file 1 [file sensors-26-02181-s001.zip › FigureS1. Scenario for Experiment2-l (HD).png]

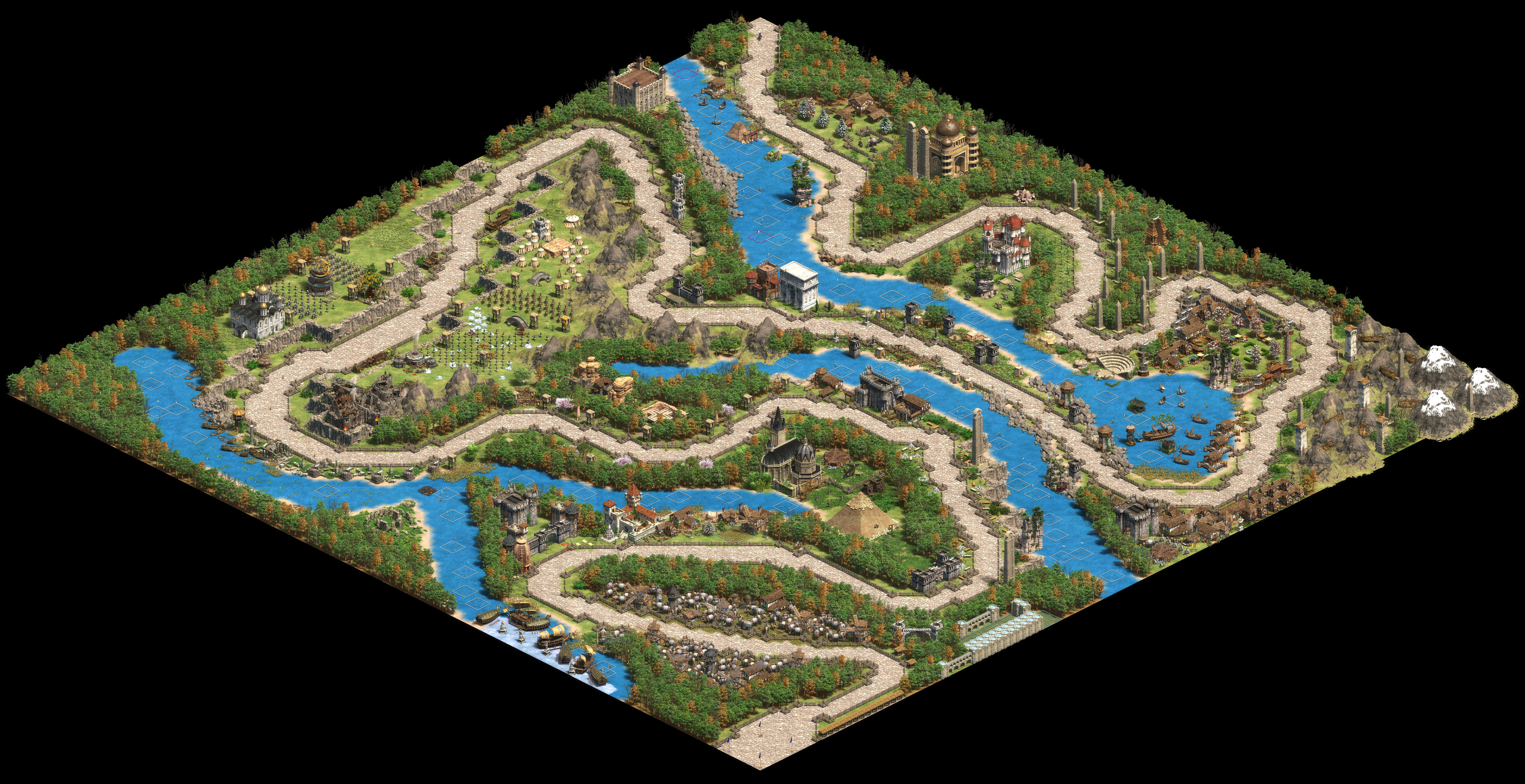

Supplement: Supplementary file 1 [file sensors-26-02181-s001.zip › FigureS2. Scenario for Experiment2-h (HD).png]

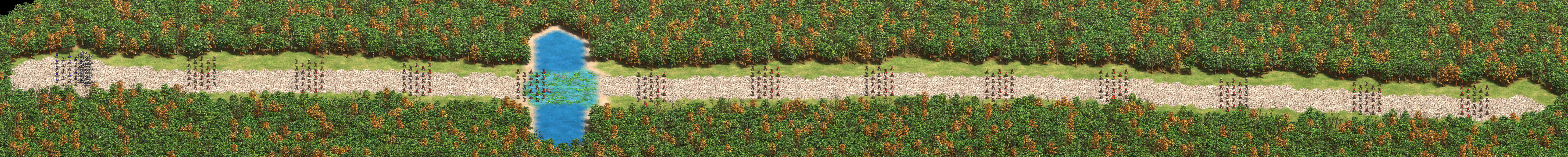

Supplement: Supplementary file 1 [file sensors-26-02181-s001.zip › FigureS3. Scenario for Experiment4 (HD).png]
